# Genetic, Epidemiological, Clinical, and Therapeutic Trajectories in Colon and Rectal Cancers

**DOI:** 10.3390/cancers17213438

**Published:** 2025-10-27

**Authors:** Maurizio Capuozzo, Carmine Picone, Francesco Sabbatino, Mariachiara Santorsola, Francesco Caraglia, Domenico Iervolino, Roberto Sirica, Oreste Gualillo, Giordana Di Mauro, Rosa Castiello, Monica Ianniello, Alessia Maria Cossu, Angela Nebbioso, Lucia Altucci, Francesco Izzo, Renato Patrone, Andrea Belli, Massimiliano Berretta, Marco Cascella, Francesco Perri, Anna Chiara Carratù, Guglielmo Nasti, Massimo Di Maio, Antonio Giordano, Giovanni Savarese, Michele Caraglia, Alessandro Ottaiano

**Affiliations:** 1 Pharmaceutical Department, Asl Napoli 3 Sud, Via Marittima 3, 80056 Ercolano, Italy; m.capuozzo@aslnapoli3sud.it; 2 Istituto Nazionale Tumori di Napoli, IRCCS “G. Pascale”, Via Mariano Semmola, 80131 Naples, Italyf.izzo@istitutotumori.na.it (F.I.); renato.patrone@istitutotumori.na.it (R.P.);; 3 Department of Medicine, Surgery and Dentistry, University of Salerno, Via Salvador Allende, 43, 84081 Baronissi, Italy; 4 Department of Precision Medicine, University of Campania “Luigi Vanvitelli”, Via Luigi De Crecchio 7, 80138 Naples, Italy; francesco.caraglia@studenti.unicampania.it (F.C.); angela.nebbioso@unicampania.it (A.N.);; 5 Clinical Statistics Department, Institute for Advanced Statistical Sciences, Via San Felice n. 16, 80036 Palma, Italy; 6 Centro AMES, Via Padre Carmine Fico 24, 80013 Casalnuovo di Napoli, Italy; 7 Laboratory of Neuroendocrine Interactions in Rheumatology and Inflammatory Diseases, IDIS, Complexo Hospitalario Universitario de Santiago de Compostela, University of Santiago de Compostela, Travesía da Choupana s/n, 15706 Santiago de Compostela, Spain; oreste.gualillo@sergas.es; 8 Department of Human Pathology “G. Barresi”, School of Specialization in Medical Oncology, Via Consolare Valeria 1, 98125 Messina, Italy; 9 Laboratory of Precision and Molecular Oncology, Biogem Scarl, Institute of Genetic Research, Contrada Camporeale, 83031 Ariano Irpino, Italy; 10 Program of Medical Epigenetics, Vanvitelli Hospital, Via Luigi De Crecchio 7, 80138 Naples, Italy; 11 Department of Clinical and Experimental Medicine, University of Messina, Via Consolare Valeria 1, 98125 Messina, Italy; mberretta@unime.it; 12 Department of Oncology, University of Turin, AOU Città della Salute e della Scienza di Torino, 10126 Turin, Italy; 13 Sbarro Institute for Cancer Research and Molecular Medicine and Center for Biotechnology, College of Science and Technology, Temple University, Philadelphia, PA 19122, USA; giordano@temple.edu; 14 Department of Medical Biotechnologies, University of Siena, 53100 Siena, Italy

**Keywords:** colorectal cancer, colon cancer, rectal cancer, cancer epidemiology, precision oncology, metastatic disease, therapeutic strategies

## Abstract

Colorectal cancer (CRC) is among the most prevalent malignancies worldwide, representing the second leading cause of cancer-related mortality and accounting for approximately 2 million new cases and nearly half a million deaths annually. Global age-standardized incidence rates are highest in Australia/New Zealand and other Western countries, and lowest in parts of sub-Saharan Africa and South Asia, reflecting variations in demographics, lifestyle exposures, and screening practices. Colon cancer constitutes the larger fraction of CRC cases, with rectal cancer contributing substantially, and early-onset CRC (<50 years) is increasing across both high-income and emerging regions. Established risk factors include age, hereditary syndromes, obesity, sedentary behavior, dietary patterns, metabolic disorders, and chronic inflammation, with notable distinctions between colon and rectal subsites. This narrative review provides a comprehensive overview of CRC epidemiology, molecular and genetic pathogenesis, staging, and modern therapeutic approaches, addressing colon and rectal cancers separately due to their distinct biology, clinical behavior, and treatment strategies. By integrating current knowledge on genetic drivers, systemic and local therapies, and patient stratification, the review aims to inform clinical practice, support clinical trial design, discuss ongoing challenges and future perspectives, and foster further research toward precision-guided management of CRC.

## Simple Summary

Colorectal cancer (CRC) is a major global health burden, ranking as the second leading cause of cancer-related deaths. Its incidence and mortality vary widely across regions, with higher rates in Australia, New Zealand, and other Western countries, and lower rates in parts of sub-Saharan Africa and South Asia. Colon and rectal cancers differ in biology, risk factors, and clinical behavior, and early-onset CRC is increasing in both high-income and emerging regions. This review provides a comprehensive overview of CRC epidemiology, molecular and genetic pathogenesis, staging, and modern therapeutic approaches, highlighting challenges in patient selection, treatment strategies, and precision oncology.

## 1. Introduction

Colorectal cancer (CRC) is one of the most prevalent and deadly malignancies worldwide. According to the Global Cancer Observatory, CRC ranks among the top three cancers in terms of incidence and mortality, with increasing rates observed in both developed and developing countries [1]. The disease arises from the progressive accumulation of genetic and epigenetic alterations in colonic epithelial cells, leading to uncontrolled proliferation, invasion, and metastasis [2]. Colorectal carcinogenesis is traditionally described by the adenoma-carcinoma sequence, a stepwise transformation of normal colonic epithelium into invasive adenocarcinoma. This process is driven by key genetic alterations, including mutations in tumor suppressor genes and oncogenes, as well as epigenetic modifications that regulate gene expression [3]. While the majority of CRC cases are sporadic, hereditary forms such as Lynch syndrome and familial adenomatous polyposis (FAP) account for a smaller subset of cases and provide valuable insights into the disease’s genetic underpinnings [4,5].

Clinically, CRC may present across a wide spectrum of manifestations, ranging from asymptomatic polyps to advanced metastatic disease. Prognosis is strongly stage-dependent: early-stage tumors are potentially curable, whereas metastatic disease generally requires systemic therapy [6]. In recent years, the advent of precision oncology has revolutionized the management of metastatic CRC, with molecular subtyping increasingly guiding therapeutic decisions. Targetable alterations such as BRAF (v-Raf murine sarcoma viral oncogene homolog B1) p.V600E mutations, HER2 (Human Epidermal Growth Factor Receptor 2) amplification, NTRK (Neurotrophic Tyrosine Receptor Kinase) fusions, and dMMR (deficient Mismatch Repair) status have reshaped therapeutic paradigms [6,7]. To this expanding repertoire of actionable molecular changes, RAS (Rat Sarcoma virus)—long considered “undruggable”—is now being reconsidered as a therapeutic target, a shift that is poised to further transform the treatment landscape [8,9].

Although our work does not specifically address anal cancer, it is important to acknowledge that squamous cell carcinoma of the anal canal and, more rarely, of the rectum represents a distinct clinicopathological entity frequently associated with persistent Human Papillomavirus (HPV) infection. HPV-driven carcinogenesis in this setting involves the development of squamous intraepithelial lesions, which may evolve toward invasive carcinoma, particularly in immunocompromised individuals [10,11].

Despite significant progress in screening and therapeutic strategies, CRC remains a major public health challenge. This review aims to trace the genetic, epidemiological, clinical, and therapeutic trajectories of the disease, providing a comprehensive and updated overview intended to advance current knowledge, guide the design of clinical trials, and support clinical decision-making, while also elucidating the key molecular players involved in the pathogenesis of colorectal neoplasms.

## 2. Most Typical Genetic Alterations in Colon Cancer: APC, p53, KRAS, SMAD4

**Figure 1:**
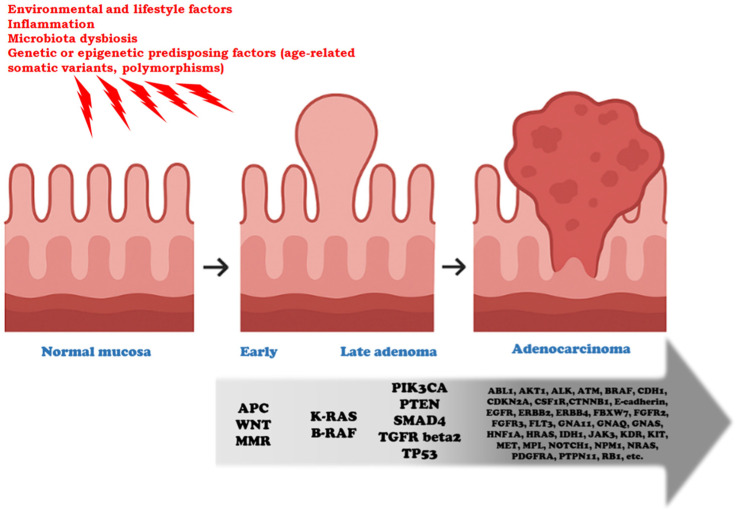
Genetic progression from early adenoma to colorectal adenocarcinoma. This model represents a conceptual framework of colorectal tumorigenesis, in which not all the listed alterations necessarily co-occur, nor should their sequence be considered dogmatic. This sequence typically occurs over 5 to 20 years. The preneoplastic normal mucosa (colonic mucosal field) is influenced by environmental, inflammatory, microbial, and genetic/epigenetic factors. These exposures act as initiating or permissive conditions that predispose the mucosa to neoplastic transformation, operating both before and during the initiation phase of tumorigenesis. Key genetic events are crucial for progression through the successive stages of neoplastic evolution. *APC* loss and Wnt/beta-catenin pathway activation drive the transition from normal mucosa to early adenoma, a stage in which defects in the DNA mismatch repair (MMR) system may also arise, leading to accelerated mutational accumulation. The acquisition of *KRAS*, *BRAF*, *PIK3CA*, *SMAD4*, or *TGFRbeta2* alterations marks progression from early to late adenoma, fostering increased proliferative and invasive potential. The emergence of *TP53* mutations, in particular, is critical for the acquisition of a fully malignant phenotype. A broad spectrum of additional driver and passenger mutations is identified in advanced adenocarcinoma (e.g., *ABL*, *AKT*, *CDH1*, *EGFR*, *ERBB2*, *FBXW7*, *FGFR2*, *KIT*, *MET*, and others), illustrating the molecular heterogeneity and clonal diversification that accompany malignant progression.

CRC exhibits a broad spectrum of genetic alterations that drive tumor initiation and progression, following a stepwise evolution from normal mucosa to adenoma and ultimately adenocarcinoma, as described by the classic Vogelstein/Fearon model [2,3,4] (Figure 1). Frequently mutated genes include APC (Adenomatous Polyposis Coli), TP53 (Tumor Protein 53), RAS, and SMAD4 (Sma and Mad—Mothers Against Decapentaplegic—related protein 4), which regulate key cellular processes such as proliferation, differentiation, and apoptosis. Alterations in these genes disrupt critical signaling pathways—including the Wnt (Wingless/Integrated)/β-catenin cascade (APC), p53-mediated DNA damage response (TP53), MAPK signaling (RAS), and TGF-β signaling (SMAD4)—promoting uncontrolled growth, genomic instability, and resistance to cell death. Understanding the roles of these driver genes provides essential insights into CRC tumorigenesis and informs the development of targeted therapies and predictive biomarkers. In addition to intrinsic genetic alterations, chronic inflammation plays a pivotal role in colorectal carcinogenesis. Pro-inflammatory cytokines, particularly interleukins such as IL-6, IL-1β, and IL-17, can induce oxidative stress and the production of reactive oxygen and nitrogen species, resulting in DNA damage that favors mutagenesis. IL-6 and IL-1β activate downstream signaling pathways, including STAT3 and NF-κB, which can promote the survival of mutated cells and enhance proliferation, thereby cooperating with classic driver mutations. Moreover, inflammatory microenvironments facilitate epigenetic modifications and genomic instability, accelerating tumor evolution [12,13,14].

### 2.1. APC

**Figure 2:**
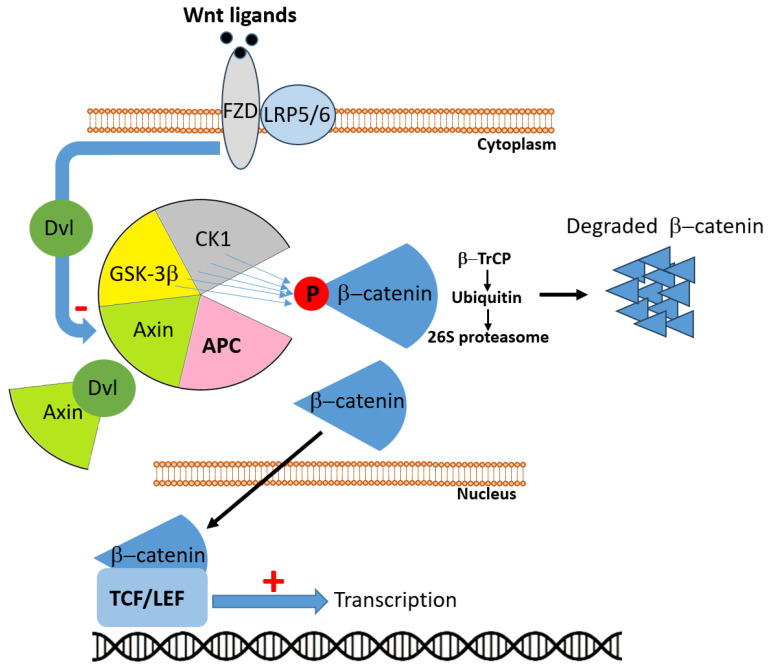
In the absence of Wnt ligands, adenomatous polyposis coli (APC) participates in the multiprotein destruction complex, composed of APC, Axin, glycogen synthase kinase 3β (GSK3β), and casein kinase 1 (CK1). Within this complex, β-catenin is sequentially phosphorylated, recognized by the E3 ubiquitin ligase β-TrCP, polyubiquitinated, and subsequently degraded by the 26S proteasome, thereby preventing its nuclear accumulation. Upon Wnt ligand binding to Frizzled (FZD) receptors and the co-receptors low-density lipoprotein receptor-related proteins 5/6 (LRP5/6), the cytoplasmic protein Dishevelled (Dvl) is activated and sequesters Axin, resulting in disassembly of the destruction complex. Consequently, β-catenin is stabilized, accumulates in the cytoplasm, and translocates into the nucleus, where it associates with T-cell factor/lymphoid enhancer factor (TCF/LEF) transcription factors to drive expression of Wnt target genes. Loss or dysfunction of APC impairs the destruction complex, leading to constitutive activation of the Wnt/β-catenin signaling pathway and uncontrolled transcriptional activity.

The *APC* gene, located on chromosome 5q21–q22, encodes a large multifunctional tumor suppressor protein (~310 kDa) that plays a pivotal role in colorectal homeostasis through regulating Wnt/β-catenin signaling, cell adhesion, migration, genomic integrity, and apoptosis. *APC* mutations—both germline in FAP and somatic in sporadic CRC—constitute a critical early event in colorectal carcinogenesis [15]. Inactivation of APC is the initiating mutation in approximately 80–85% of sporadic CRCs, marking the onset of the adenoma–carcinoma sequence [16]. In physiological conditions, APC represents a critical partner in the Wnt signaling pathway. In the absence of Wnt ligands, APC participates in the cytoplasmic destruction complex, together with AXIN (Axis Inhibition protein), GSK3β (Glycogen Synthase Kinase 3 beta), and CK1 (Casein Kinase 1), where it promotes the stepwise phosphorylation of β-catenin (Figure 2). This modification targets β-catenin for ubiquitination and proteasomal degradation, thereby preventing its nuclear accumulation and the transcription of Wnt target genes. By sustaining this mechanism, APC exerts a fundamental role in maintaining tissue homeostasis and controlling cell proliferation and differentiation [16,17]. When Wnt ligands bind to the FZD (Frizzled) receptor and its co-receptor LRP5/6(Low-Density Lipoprotein Receptor-Related Protein 5/6), the signal is transduced via Dvl (Dishevelled), which sequesters AXIN at the membrane and destabilizes the destruction complex. Under these conditions, the APC-driven degradation of β-catenin is inhibited, leading to β-catenin stabilization, cytoplasmic accumulation, and nuclear translocation. Within the nucleus, β-catenin associates with TCF (T-cell factor)/LEF (Lymphoid Enhancer-binding Factor) transcription factors to activate genes involved in proliferation, survival, and stemness. By contrast, loss or inactivation of APC impair the integrity of the destruction complex, uncoupling β-catenin degradation from Wnt receptor stimulation. As a result, β-catenin constitutively accumulates in the nucleus, where it drives persistent activation of Wnt target genes, fueling uncontrolled cell division and malignant transformation [17].

Beyond its canonical role, APC also exerts non-Wnt functions that contribute to its tumor-suppressive capacity, including maintenance of chromosomal stability, regulation of microtubule dynamics, DNA repair, cell adhesion, and modulation of the tumor microenvironment (TME) [18]. The clustering of truncating mutations in the mutation cluster region (MCR) of APC frequently results in protein truncations that abrogate its regulatory domains and are seen ubiquitously in CRC [19].

The dual-hit nature of APC inactivation (biallelic mutations) follows the Knudson model, where FAP patients harbor a germline mutation and develop polyps that, upon acquiring a second somatic hit, rapidly progress to carcinoma [15]. Intriguingly, emerging quantitative models suggest that incomplete APC inactivation—rather than complete loss—may confer an optimal “just-right” level of Wnt signaling that promotes tumorigenesis more effectively than total disruption [20].

Moreover, recent prognostic studies indicate that the mutation status of APC holds prognostic value: tumors with two truncating APC mutations, especially in combination with *KRAS* (Kirsten Rat Sarcoma viral oncogene homolog) and *TP53* alterations, are associated with poorer survival outcomes; conversely, tumors lacking any APC mutation may follow distinct, non-Wnt driven malignant routes [16].

Therapeutic approaches targeting APC/Wnt signaling are currently in development but remain largely indirect, given the difficulty of restoring tumor-suppressor function. Strategies include small-molecule inhibitors and biologics that target downstream effectors of Wnt signaling, such as porcupine inhibitors (e.g., LGK974) that block Wnt ligand secretion, tankyrase inhibitors that destabilize β-catenin [21], and β-catenin/TCF interaction antagonists [22]. Other approaches aim to exploit synthetic-lethal vulnerabilities in APC-deficient cells, including modulators of DNA repair, metabolic dependencies, or cell-cycle checkpoints [23]. Although clinical translation is still in early stages, these strategies offer the potential to selectively target APC-mutant CRC and mitigate Wnt-driven tumor progression.

### 2.2. TP53

**Figure 3:**
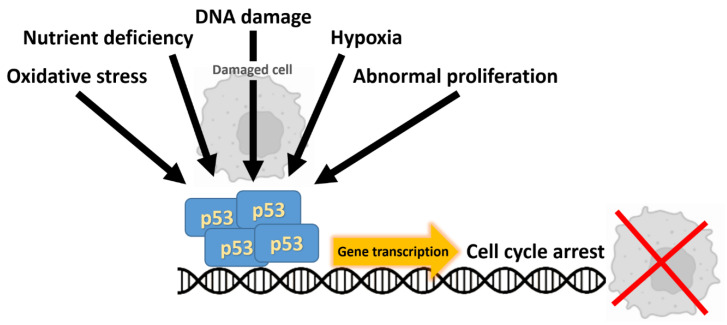
The tumor suppressor p53 is activated by a variety of cellular stressors. Upon activation, p53 forms a tetramer that coordinates downstream responses by driving gene transcription, ultimately resulting in cell cycle arrest or the induction of apoptosis.

*TP53*, located on chromosome 17p13, encodes the p53 transcription factor, a central coordinator of the cellular response to genotoxic stress that enforces cell-cycle arrest, DNA repair, senescence and apoptosis (Figure 3) [24]. In colorectal carcinogenesis, loss of p53 function is typically a relatively late event in the adenoma–carcinoma sequence and commonly coincides with transition to invasive carcinoma, a cornerstone of the classic Vogelstein/Fearon genetic progression model [2]. Somatic *TP53* alterations are among the most frequent genetic lesions in CRC, reported in roughly 40–60% of tumors depending on cohort and assay, with a predominance of missense substitutions clustered in DNA-binding “hotspot” residues [24]. Many of these missense mutants not only abrogate wild-type tumor-suppressor activities but can exert dominant-negative effects or acquire oncogenic “gain-of-function” properties that promote invasion, metastatic dissemination and altered transcriptional programs—phenomena that have been linked to distinct clinical behaviors according to the specific mutant allele (for example, R175 vs. R273 substitutions) [25]. Clinically, TP53 mutation status has been associated with adverse outcomes across several tumor types; however, data in CRC remain inconsistent and sometimes contradictory [26].

Given this biological and clinical complexity, current translational research is directed toward: (i) high-resolution characterization of TP53 allelic status and mutation type in well-annotated cohorts; (ii) functional stratification of mutants into loss-of-function, dominant-negative, or gain-of-function categories; and (iii) development of therapeutic strategies aimed at restoring wild-type p53 activity, exploiting synthetic-lethal vulnerabilities in p53-deficient tumors, or counteracting oncogenic pathways driven by mutant *TP53*. Collectively, these approaches seek to transform *TP53* from a descriptive biomarker into an actionable determinant for patient stratification and therapeutic intervention [26].

### 2.3. RAS

**Figure 4:**
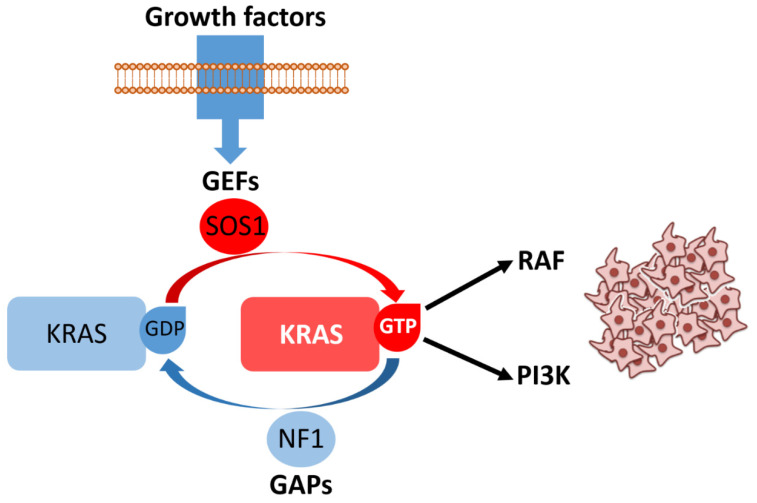
Growth factor binding to receptor tyrosine kinases promotes recruitment of guanine nucleotide exchange factors (GEFs), such as son of sevenless homolog 1 (SOS1), which catalyze the conversion of KRAS bound to guanosine diphosphate (KRAS/GDP, inactive state) into KRAS bound to guanosine triphosphate (KRAS/GTP, active state). The intrinsic GTPase activity of KRAS is normally stimulated by GTPase-activating proteins (GAPs), including neurofibromin 1 (NF1), ensuring timely signal termination. In its GTP-bound form, KRAS activates key effectors such as rapidly accelerated fibrosarcoma (RAF) kinases and phosphoinositide 3-kinase (PI3K), driving downstream signaling cascades that promote cell proliferation and survival. Aberrant or persistent KRAS activation results in uncontrolled proliferative signaling, illustrated here by the accumulation of neoplastic cells.

KRAS and NRAS (Neuroblastoma RAS viral oncogene homolog) encode closely related small GTPases (Guanosine Triphosphatases) that function as binary molecular switches within the RAS–RAF (Rapidly Accelerated Fibrosarcoma)–MEK (Mitogen-Activated Protein Kinase Kinase)–ERK (Extracellular Signal-Regulated Kinase) mitogen-activated protein kinase (MAPK) cascade and additionally modulate PI3K (Phosphatidylinositol 3-Kinase)–AKT (Protein Kinase B) signaling (Figure 4). In their GTP-bound state, they adopt an active conformation that recruits downstream effectors, whereas oncogenic point mutations lock RAS in this constitutively active form, decoupling signaling from upstream receptor control and promoting mitogenic and pro-survival programs [27]. Mutational activation of KRAS is among the most frequent molecular events in CRC, detected in approximately one-third to two-fifths of unselected cases (commonly ~40%), with hotspot substitutions predominantly at codons 12 and 13, and a smaller but clinically relevant subset at codon 61. The exact prevalence shows modest variation between cohorts and according to tumor stage and geography [28]. By contrast, *NRAS* mutations—although functionally similar in terms of their cellular effects—are much less common, occurring in only ~2–5% of large series [29]. From a clinical standpoint, activating *RAS* mutations have two major consequences. First, *RAS* mutational status is a well-established predictive biomarker for lack of benefit from anti-EGFR (Epidermal Growth Factor Receptor) monoclonal antibodies (cetuximab, panitumumab): tumors carrying constitutively active *KRAS* or *NRAS* signal downstream of EGFR and are therefore independent of receptor activation, explaining the primary resistance observed in randomized trials and meta-analyses that established the need for extended *RAS* testing prior to anti-EGFR therapy [30]. Second, *RAS* mutations are independently associated with unfavorable prognosis in several cohorts and meta-analyses, although the strength of this effect varies with the specific allele (e.g., G12C, G12V) and disease context [31,32].

Mechanisms of acquired resistance are also frequently *RAS*-driven: under the selective pressure of anti-EGFR therapy, subclonal *RAS* mutations or gene amplifications can arise (detectable in tissue or circulating tumor DNA–ctDNA–), producing secondary resistance even in tumors that were *RAS* wild-type at baseline. This adaptive evolution underscores the importance of serial molecular monitoring and informs the design of combinatorial strategies aimed at delaying or overcoming resistance [33]. This topic will also be addressed in more detail later in this review.

Finally, the long-held perception of *KRAS* as “undruggable” has been challenged by the development of allele-specific inhibitors (most notably targeting *KRAS* p.G12C) and by ongoing efforts to exploit downstream dependencies or combine KRAS inhibitors with EGFR, MEK, or immune-modulating agents. These advances have generated a rapidly evolving therapeutic landscape in which precise *RAS* genotyping—including codon- and allele-level annotation—has become essential for patient selection and clinical trial design [8,9]

### 2.4. SMAD4

**Figure 5:**
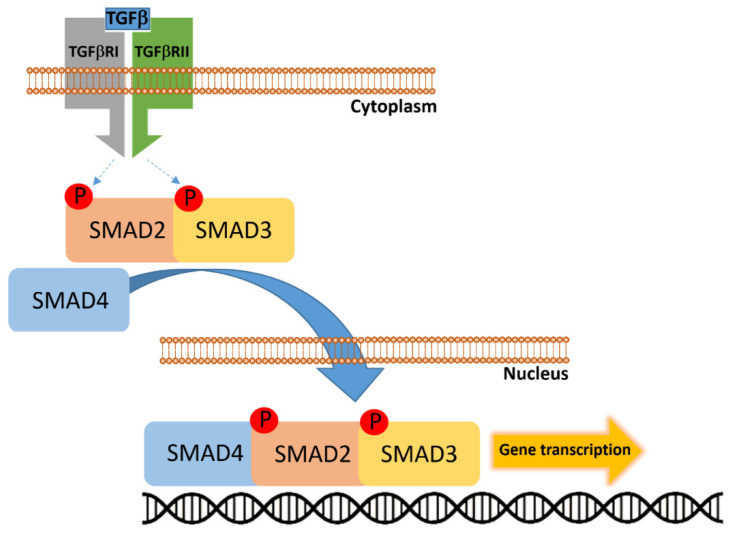
Transforming growth factor-β (TGF-β) binds to the heterotetrameric receptor complex composed of TGF-β receptor type I (TGFBRI) and type II (TGFBRII), leading to phosphorylation and activation of receptor-regulated SMADs (R-SMADs), primarily SMAD2 and SMAD3. These phosphorylated SMADs then associate with the common-mediator SMAD4 to form a heteromeric complex. The SMAD2/3–SMAD4 complex translocates into the nucleus, where it regulates transcription of target genes involved in cell cycle control, differentiation, and tumor suppression.

*SMAD4*, located at chromosome 18q21, encodes the Co-SMAD (common-mediator SMAD) that integrates receptor-regulated SMAD signals downstream of the TGF-β (Transforming Growth Factor Beta) superfamily and transduces them to the nucleus to regulate transcriptional programs governing cell cycle arrest, differentiation, and apoptosis (Figure 5) [34,35]. Somatic inactivation of SMAD4—by mutation, homozygous deletion, or loss of protein expression—is recurrent in CRC, with reported frequencies ranging broadly (commonly ~10% in large cohorts, with study-to-study variability up to ~20% depending on assay and stage) and is enriched in advanced-stage and metastatic disease [36]. Functionally, loss of *SMAD4* abrogates canonical TGF-β–mediated growth inhibition and cell-cycle arrest, shifting the net effect of the TGF-β axis from tumor suppression toward tumor-promoting processes such as EMT (Epithelial–Mesenchymal Transition), invasion, and stromal remodeling—mechanisms that plausibly underpin the association between *SMAD4* loss and worse overall survival and therapeutic resistance in CRC patients [37]. Mechanistic studies have begun to define specific downstream effectors through which *SMAD4* deficiency promotes dissemination: for example, *SMAD4* loss induces CCL15 (Chemokine C-C motif Ligand 15) expression and consequent recruitment of CCR1+ (C-C Chemokine Receptor type 1 positive) myeloid cells that facilitate hepatic colonization, providing a molecular explanation for the frequent link between SMAD4 loss and liver metastasis [38,39]. Parallel work shows that SMAD4 deficiency can reprogram BMP (Bone Morphogenetic Protein)/TGF-β family signaling and interact with MAPK/ERK, c-MYC (cellular MYelocytomatosis oncogene), and ribosome-biogenesis programs (e.g., NLE1–Notchless Homolog 1–upregulation) to sustain proliferative and stem-like phenotypes in organoid and in vivo models—pathways that both explain aggressive biology and suggest targetable vulnerabilities [40,41]. Therapeutically, *SMAD4* loss is being explored both as a prognostic/predictive biomarker and as a means to guide targeted interventions. Strategies under investigation include: (i) modulation of the broader TGF-β axis (ligand traps, receptor kinase inhibitors, or antisense approaches) to blunt pro-metastatic TGF-β signaling where appropriate; (ii) targeting SMAD4-synthetic lethal partners or downstream effectors (e.g., inhibition of PI3K/AKT/mTOR (mechanistic Target of Rapamycin) or RICTOR (Rapamycin-Insensitive Companion of mTOR) in SMAD4-negative tumors) that sensitize cells to chemotherapy or impair metastatic fitness; and (iii) intercepting stromal/immune recruitment axes (for example, by blocking the CCL15–CCR1 chemokine axis) to prevent creation of a permissive metastatic niche [38,42,43].

## 3. Additional Actionable Alterations in CRC: BRAF and HER2

### 3.1. BRAF

*BRAF* alteration cannot be regarded as a typical or frequent event in CRC; however, it deserves particular attention due to its significant therapeutic implications. The prevalence of *BRAF* mutations in CRC is generally reported to be approximately 5% in unselected cohorts, with the p.V600E substitution accounting for the vast majority of cases. These mutations are characteristically enriched in right-sided tumors, in older female patients, and in tumors exhibiting high levels of microsatellite instability (MSI-H) [44,45]. *BRAF* encodes a serine/threonine kinase that occupies a central node of the RAS–RAF–MEK–ERK (MAPK) signaling cascade and thereby regulates cellular proliferation, differentiation and survival [46].

Clinically, *BRAF* p.V600E defines a biologically and prognostically distinct CRC subgroup: it is associated with aggressive behavior, a higher propensity for peritoneal and distant spread, and inferior overall survival compared with *BRAF*-wildtype disease, particularly in the metastatic setting. Moreover, *BRAF* p.V600E frequently co-occurs with specific histologic and molecular features (for example, mucinous histology and MSI in a subset) that influence therapeutic response and trial design [47].

Mechanistic studies explain why single-agent *BRAF* inhibitors—highly effective in melanoma—produce only limited activity in CRC: feedback reactivation of upstream EGFR signaling and rapid MAPK pathway re-engagement (via RAS activation or alternative RAF dimerization) circumvent RAF blockade, resulting in primary resistance in many *BRAF*-mutant CRC. These observations provided the rationale for combinatorial strategies that simultaneously suppress BRAF and block EGFR (and, in some trials, downstream MEK), and for exploring additional co-targets that arise from acquired resistance mechanisms (e.g., *RAS* alterations, *EGFR* ectodomain mutations, or MAP2K/ERK pathway reactivations) [48].

Therapeutically, the clinical development of BRAF–directed combinations has changed the treatment landscape for patients with *BRAF* p.V600E metastatic CRC. The randomized phase III BEACON (BRAF, EGFR, and MEK inhibition in BRAF V600E-mutant CRC) trial demonstrated that the combination of a selective BRAF inhibitor with an EGFR antibody (encorafenib plus cetuximab), with or without a MEK inhibitor, improved response rates and overall survival compared with standard chemotherapy in previously treated patients, establishing targeted BRAF + EGFR inhibition as a standard option in the refractory setting [49]. Subsequent work has extended this approach into earlier lines: recent randomized data show that first-line EC (encorafenib + cetuximab) with chemotherapy (EC + modified FOLFOX6 [mFOLFOX6: leucovorin, 5-fluorouracil, and oxaliplatin]) yields superior progression-free and overall survival compared with standard regimens in untreated *BRAF* p.V600E metastatic CRC, supporting the movement of *BRAF*-targeted combinations into front-line therapy for selected patients [50].

### 3.2. HER2

HER2 (human epidermal growth factor receptor 2), encoded by the *ERBB2* (erb-b2 receptor tyrosine kinase 2) gene on chromosome 17q12, is a transmembrane tyrosine kinase receptor that under physiological conditions regulates cell growth, differentiation, and survival. Similarly to BRAF, HER2 does not represent a canonical alteration in CRC, as HER2 aberrations—including gene amplification or activating mutations—occur in fewer than5% of cases. These events appear more frequently in male patients and in Asian and Black populations [51]. These alterations are associated with aggressive tumor behavior and poor prognosis [52]. Functionally, HER2 overexpression leads to the activation of downstream signaling pathways, including MAPK and PI3K/AKT pathways, promoting tumor cell proliferation and survival. This aberrant signaling contributes to tumor progression and metastatic dissemination, while also driving resistance to anti-EGFR therapies. Notably, concurrent mutations in other oncogenes, such as *KRAS*, *NRAS*, and *PIK3CA* (phosphatidylinositol-4,5-bisphosphate 3-kinase catalytic subunit alpha), have been identified in HER2-amplified CRCs, although their exact role in resistance mechanisms remains under investigation [53].

Therapeutically, HER2 represents a viable target in metastatic CRC. The combination of trastuzumab (Herceptin) and tucatinib (Tukysa) has been approved by the FDA (Food and Drug Administration) for the treatment of HER2-positive, *RAS* wild-type metastatic CRC following prior chemotherapy, based on the results of the MOUNTAINEER trial [54].

Although *APC*, *TP53*, *KRAS*, and *SMAD4* are traditionally regarded as the principal genetic drivers of colorectal carcinogenesis, this framework represents only a partial view of a far more intricate biological landscape. Indeed, subsets of CRCs lacking such canonical alterations clearly exist, indicating that alternative oncogenic routes and compensatory molecular networks can sustain malignant transformation. This observation highlights the plasticity and evolutionary adaptability of colorectal tumors, where convergent phenotypes may arise from divergent genotypes. Many oncogenic drivers likely remain elusive—masked within the “long tail” of low-frequency or context-dependent events, or embedded within noncoding, epigenetic, and transcriptomic layers of regulation. Future research integrating multi-omic, spatial, and temporal analyses will therefore be essential to move beyond the historical paradigm of a few dominant genes and toward a more complete, systems-level understanding of colorectal tumorigenesis.

## 4. Distinct Genomic and Microenvironmental Trajectories in Colon Versus Rectal Cancer

Embryologically, the proximal (right) colon (cecum, ascending colon and proximal two-thirds of the transverse colon) derives from the midgut whereas the distal (left) colon (distal one-third of the transverse colon, descending colon and sigmoid colon) and the rectum derive from the hindgut; these developmental origins underpin later molecular and microenvironmental divergence [55]. At the level of early neoplastic progression many colorectal tumors share canonical initiating events of the adenoma–carcinoma sequence (for example, alterations of *APC*, early *KRAS* activation and subsequent *TP53* inactivation), indicating a broadly conserved “early” genetic program across colon and rectum [56]. However, in established disease the molecular landscapes diverge markedly. Right-sided colon cancers are disproportionately enriched for MSI-H, CpG island methylator phenotype (CIMP), higher overall mutational burden and higher frequency of *BRAF* p.V600E and certain *PIK3CA* mutations, and are more often associated with immune/“inflammatory” transcriptional programs (the CMS1/immune-high group) [57,58]. By contrast, left-sided colon and rectal tumors show greater prevalence of chromosomal instability (CIN) and somatic copy-number alterations, higher rates of *TP53* mutations and specific recurrent CNV-driven oncogenic events (and hence tend to map more frequently to the CMS2 “canonical” epithelial subgroup), while a subset of tumors in distal sites display mesenchymal/stromal signatures (CMS4) with prominent desmoplastic features [59,60]. Rectal cancers—although they arise through the same early APC/KRAS/TP53 pathway—exhibit a distinctive final repertoire of alterations and proteomic hubs compared with proximal colon tumors: rectal primaries have been reported to show relatively higher *TP53* and *FBXW7* mutation frequencies, higher rates of TOPO1 expression and a distinct pattern of *ERBB2* amplification/mutation and other targetable alterations that differ from both left and right colon in frequency and hotspot distribution [57,58,59,60,61]. The TME and local microbiome also differ by site: proximal tumors with MSI-H tend to be immune-infiltrated, whereas distal (including rectal) tumors show different immune cell compositions, stromal/CAF (cancer-associated fibroblast) enrichment and characteristic microbial signatures that may influence mutational selection and response to therapy [61,62]. The distinctive biological characteristics of colorectal cancer according to tumor location are summarized in Table 1.

Taken together, these data indicate that, beyond shared early drivers, rectal cancer frequently converges on molecular-genetic and TME states that are distinct from right- and left-sided colon cancers; these differences have clear implications for stratification, biomarker development and location-specific therapeutic strategies [63].

**Table 1:** Distinct genomic, microenvironmental, and microbial features of right-sided, left-sided, and rectal cancers.

Feature	Right-Sided Colon Cancer	Left-Sided Colon Cancer	Rectal Cancer
Embryologic origin	Midgut (cecum, ascending, proximal 2/3 transverse colon)	Hindgut (distal 1/3 transverse, descending, sigmoid)	Hindgut
Molecular phenotype	Enriched for MSI-H, CIMP, higher mutational burden	Predominantly CIN, frequent somatic copy-number alterations	Higher TP53 and FBXW7 mutation frequencies
Key mutations/alterations	*BRAF* p.V600E, certain *PIK3CA* mutations	High *TP53* mutation rate, recurrent CNV-driven oncogenic events	*TP53*, *FBXW7* mutations; higher TOPO1 expression and *ERBB2* alterations
Transcriptomic CMS subtypes	More frequent CMS1 (immune-high)	More frequent CMS2 (“canonical” epithelial); subset CMS4 (mesenchymal/stromal, desmoplastic)	More frequent CMS2; enrichment for CMS4 in post-neoadjuvant resection specimens
Tumor microenvironment	Immune-infiltrated (particularly in MSI-H)	Variable immune composition; subset enriched in stromal/desmoplastic signatures (CMS4)	Stromal/CAF enrichment
Microbiome	Fusobacterium dominance	Bacteroides/Desulfovibrio shift	Bacteroides/Desulfovibrio shift

CAF, cancer-associated fibroblast; CIN, chromosomal instability; CIMP, CpG island methylator phenotype; CMS, consensus molecular subtype; CNV, copy number variation; ERBB2, erb-b2 receptor tyrosine kinase 2; FBXW7, F-box and WD repeat domain containing 7; MSI-H, microsatellite instability-high; PIK3CA, phosphatidylinositol-4,5-bisphosphate 3-kinase catalytic subunit alpha; TME, tumor microenvironment; TOPO1, topoisomerase I; TP53, tumor protein p53.

## 5. Epidemiology of Colon and Rectal Cancers

CRC arises from a complex interplay between genetic susceptibility, environmental exposures, and lifestyle factors, with notable differences between colon and rectal cancer. Established risk factors include age, family history, and hereditary syndromes such as Lynch syndrome and FAP [64]. Lifestyle determinants, including obesity, sedentary behavior, alcohol consumption, and tobacco use, significantly contribute to CRC incidence worldwide [65]. Dietary habits exert a pivotal role: high intake of red and processed meat is associated with increased risk, whereas fiber-rich diets and regular consumption of fruits and vegetables exert protective effects [66]. Metabolic conditions such as type 2 diabetes and chronic inflammation, particularly inflammatory bowel diseases, further elevate CRC risk [67,68,69,70]. Importantly, distinctions between colon and rectal cancer are emerging: obesity and diabetes appear more strongly associated with colon cancer, while alcohol and smoking show a tighter link with rectal cancer [71,72,73]. CRC remains one of the most important contributors to the global cancer burden (Table 2).

In 2020, approximately 1.9–1.93 million new CRC cases and roughly 0.9–0.94 million deaths occurred worldwide, making it among the three most frequently diagnosed cancers and the second leading cause of cancer mortality globally [74,75]. However, age-standardized incidence and mortality rates vary markedly between world regions: the highest incidence rates are observed in Australia/New Zealand and in several European and USA populations, whereas the lowest rates are recorded in parts of sub-Saharan Africa and South Asia; mortality shows similar geographic heterogeneity with particularly high rates in Eastern Europe [75]. Regional and continental patterns reflect a combination of demographic change, risk-factor prevalence and the availability of screening and treatment. High-income regions that implemented organized screening and have long-standing population-level risk-factor control have generally seen stabilization or declines in age-standardized incidence and substantial mortality reductions over recent decades; however, because these regions started from a high baseline incidence, absolute case counts remain large [76]. Conversely, many low- and middle-income countries (LMICs) currently undergoing rapid economic development and “westernization” of diet and lifestyle are experiencing rising CRC incidence, a trend attributed to changes in obesity prevalence, dietary patterns (increased processed/red meat intake), sedentary behavior and other modifiable exposures [75,76]. These transitions are accompanied by shifts in the gut microbiome composition toward pro-inflammatory and genotoxic bacterial species (such as Fusobacterium nucleatum, Bacteroides fragilis, and Escherichia coli strains producing colibactin), which may synergize with high-fat, low-fiber diets to promote mucosal inflammation, DNA damage, and carcinogenic metabolic profiles [77,78]. Therefore, the epidemiologic transition of CRC in developing countries is not merely a reflection of Western habits but represents a deeper biological shift in host–microbiome interactions and metabolic homeostasis. Furthermore, in recent years, additional attention has focused on the dietary transition occurring in many countries traditionally adhering to the Mediterranean diet [79,80]. The progressive abandonment of this dietary model—characterized by abundant consumption of vegetables, fruits, whole grains, and legumes, moderate intake of fish and olive oil, and minimal consumption of red or processed meat—has been associated with unfavorable metabolic and oncogenic consequences. Legumes, once regarded as “poor man’s proteins,” provide fiber, phytoestrogens, and bioactive compounds with anti-inflammatory and anti-proliferative properties that contribute to intestinal homeostasis. Their reduced intake, together with increased consumption of saturated fats and processed foods, has profound implications for the gut microbiota composition, leading to dysbiosis characterized by a higher Firmicutes/Bacteroidetes ratio, enrichment in pro-inflammatory bacterial species, and diminished production of short-chain fatty acids such as butyrate. These microbiome shifts alter mucosal immunity and promote genotoxic and epigenetic modifications in colonocytes, thereby facilitating tumor initiation and progression through microbe–host metabolic and signaling interactions [81].

**Figure 6:**
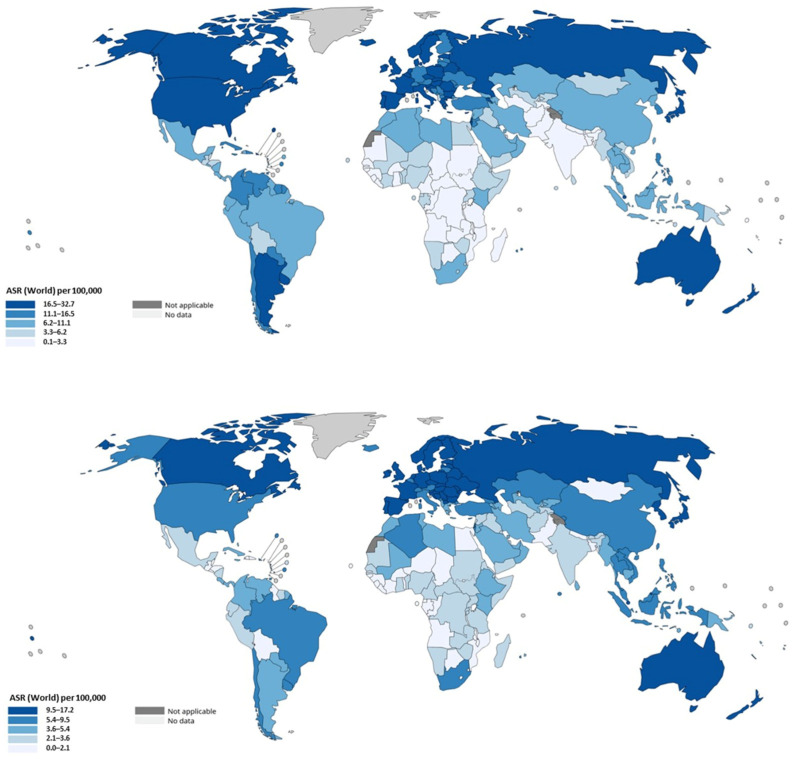
Age-standardized incidence rates of colon (upper panel) and rectal (lower panel) cancer by region. Data were extracted from the publicly available tool of the International Agency for Research on Cancer (IARC) of the World Health Organization (WHO) (https://gco.iarc.fr/today/en; last accessed 25 September 2025).

It is clinically and epidemiologically informative to separate colon cancer from rectal cancer, because they differ in risk patterns, screening detectability, and temporal trends. Global estimates derived from cancer registries and GLOBOCAN data indicate that colon cancers constitute the larger fraction of CRC burden (on the order of ~1.1–1.14 million colon cancer cases in recent GLOBOCAN reports) whereas rectal cancer accounts for several hundred thousand additional cases; together they compose the total CRC burden but with important differences by region and age. In high-income settings there has been a relative shift toward left-sided tumors and an increase in the proportional incidence of rectal cancers in some populations; in the United States, for example, the proportion of rectal tumors among CRC cases increased modestly across recent decades [82]. The incidence rates are presented in Figure 6.

Age-specific trends have attracted increasing attention because of a demonstrable rise in CRC incidence among younger adults (commonly defined as <50 years). Multiple analyses of registry data show rising early-onset CRC incidence in many countries since the 1990s, even as incidence among older, screening-eligible adults has stabilized or decreased in places with high screening coverage [83]. The early-onset increase appears to affect both colon and rectal subsites, although some reports suggest particularly rapid rises in rectal cancer incidence among young adults in certain countries; proposed contributors include obesity and metabolic dysregulation, changes in the gut microbiome, and early-life exposures, but causal mechanisms remain incompletely defined [84,85]. Emerging evidence suggests that early-life nutritional patterns, antibiotic exposure, and perinatal microbiome imprinting may alter immune tolerance and epithelial homeostasis, predisposing to carcinogenesis decades later [86,87]. These findings underscore the need for integrated life-course approaches to prevention, including maternal–child dietary education and prudent antibiotic stewardship.

Projections based on demographic change and current incidence patterns predict a substantial increase in absolute CRC case numbers by 2040—estimates indicate an increase to ~3.1–3.2 million new cases and up to ~1.6 million deaths annually if current trends and population aging proceed without further mitigation [84,85]. The projected burden will fall disproportionately on countries with growing and aging populations and on regions in epidemiologic transition unless primary prevention and high-quality screening are scaled up. From a public health perspective, these data highlight the urgency of integrating dietary and microbiome-targeted interventions (e.g., promoting fiber-rich and plant-based diets, reducing processed meat consumption, and supporting probiotic/prebiotic strategies) into national cancer prevention programs, alongside equitable access to colonoscopic and non-invasive screening tools. Without such comprehensive interventions, the global disparity in CRC burden is expected to widen, particularly among younger and socioeconomically disadvantaged populations.

## 6. Clinical Manifestations of Colon Cancer: From Polyps to Adenocarcinoma and Stages I–IV

### 6.1. Polyps and Progression to Adenocarcinoma

CRC progresses through a well-defined sequence of clinical and morphological changes, starting from benign precursor lesions and culminating in invasive carcinoma. It has become a paradigmatic model for studying the genetic and morphological evolution of cancer. In fact, most CRC cases originate from precancerous polyps, including adenomatous and serrated polyps [88]. Adenomas, particularly those with high-grade dysplasia, are at an increased risk of progression to invasive adenocarcinoma. Serrated lesions, including sessile serrated adenomas, also exhibit malignant potential, especially in cases with hypermethylation-driven *BRAF* mutations. The transition from an adenomatous polyp to invasive adenocarcinoma typically occurs over 10–15 years, driven by the progressive accumulation of genetic alterations [89]. The progression from normal colonic mucosa to adenocarcinoma represents one of the best-characterized models of stepwise cancer evolution, offering valuable insights into early carcinogenesis [90]. As previously noted, this multistep process, commonly referred to as the adenoma–carcinoma sequence, is characterized by the progressive accumulation of genetic and epigenetic alterations that drive phenotypic changes in epithelial cells [91]. Initially, mutations in the *APC* gene—an early gatekeeper—disrupt Wnt/β-catenin signaling and promote clonal expansion of aberrant crypt foci, the earliest histological lesion [92]. These foci evolve into small tubular adenomas, characterized by low-grade dysplasia. As the lesion enlarges, further mutations frequently affect oncogenes such as *KRAS*, enhancing proliferative signaling and supporting the transition to more advanced adenomas with high-grade dysplasia [93]. Disruption of additional pathways, including *TP53* inactivation and loss of chromosomal integrity, contributes to malignant transformation, culminating in invasive adenocarcinoma [94]. The TME co-evolves with these changes, facilitating angiogenesis, immune evasion, and stromal remodeling [95]. Importantly, this sequence underscores the role of intratumoral heterogeneity and clonal competition even at pre-invasive stages [96]. Emerging evidence also indicates that non-genetic mechanisms—including epigenetic reprogramming, metabolic rewiring, and stem-like cell plasticity—further contribute to the transition from benign to malignant states [97]. This continuum, observable in both sporadic CRC and syndromic contexts such as FAP, exemplifies how cancer arises from a Darwinian process of somatic evolution [98]. As such, the adenoma–carcinoma sequence remains a critical paradigm for studying tumor initiation, progression, and for refining early detection and prevention strategies [99]. Once the basement membrane is breached, the lesion is histologically classified as invasive adenocarcinoma [100].

### 6.2. Clinical Manifestations of Polyps and Non-Metastatic Disease

**Figure 7:**
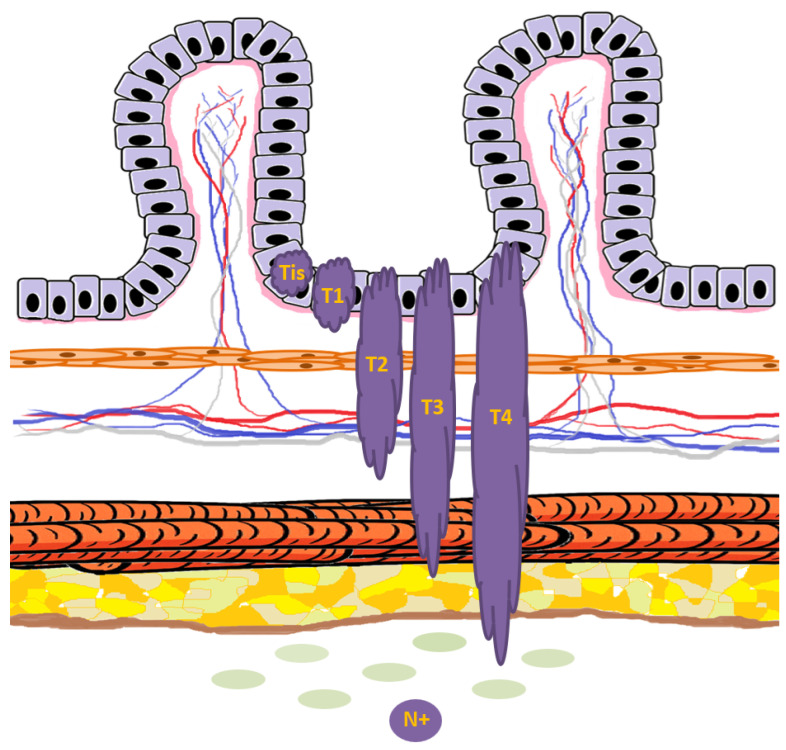
Schematic representation of T and N colorectal cancer staging in relation to the normal mucosal layers. Tumor lesions are depicted in dark purple, while the basement membrane is shown in light pink. Tis indicates carcinoma in situ, confined to the epithelium and not breaching the basement membrane. T1 represents invasion through the muscularis mucosae into the submucosa. T2 denotes invasion into, but not beyond, the muscularis propria. T3 indicates tumor extension through the muscularis propria into the subserosa or pericolorectal tissue. T4 is defined by direct tumor penetration of the visceral peritoneum (T4a) or invasion of adjacent organs/structures (T4b). The presence of tumor spread to regional lymph nodes defines N+.

The clinical presentation of colorectal polyps and non-metastatic adenocarcinoma exhibits considerable variability, ranging from asymptomatic lesions to markedly symptomatic disease [101]. Epidemiological studies indicate that the majority of colorectal polyps, particularly small tubular adenomas, remain clinically silent and are often detected incidentally during screening colonoscopy or imaging studies [102]. Similarly, early-stage adenocarcinomas—corresponding to Tis (carcinoma in situ), T1, and T2 lesions—frequently present without overt symptoms, emphasizing the critical role of early detection strategies (Figure 7) [103]. Symptomatic presentation typically emerges as the lesion enlarges or infiltrates deeper layers of the colonic wall. The most common clinical features include overt or occult gastrointestinal bleeding, detectable as melena or hematochezia, and alterations in bowel habits such as diarrhea, constipation, or changes in stool caliber [104]. In some cases, particularly with large polyps, patients may experience significant discomfort or anemia despite the absence of invasive malignancy, illustrating that polyp size does not strictly correlate with histological stage [105]. Conversely, small adenocarcinomas with limited local invasion may be asymptomatic, whereas T3 and T4 tumors—more likely associated with loco-regional lymph node involvement—exhibit an increased frequency of symptomatic manifestations, reflecting both tumor bulk and local inflammatory or obstructive effects (Figure 7) [106]. In more advanced pre-metastatic stages, patients may experience sub-occlusive episodes or frank bowel obstruction, though complete occlusion remains uncommon in early non-metastatic disease [107]. The overlap in clinical manifestations between large polyps and early adenocarcinomas underscores the challenge of relying solely on symptomatology for diagnosis, and highlights the necessity for structured screening programs. Population-based screening using fecal immunochemical testing (FIT), stool DNA testing, and colonoscopy has demonstrated efficacy in detecting both advanced adenomas and early-stage cancers, thereby enabling timely intervention before progression to invasive or metastatic disease [108,109]. Early identification of high-risk lesions, irrespective of symptomatic status, remains a cornerstone of CRC prevention, emphasizing that both polypoid and small invasive lesions constitute critical targets for surveillance and therapeutic intervention [110]. The clinical continuum observed—from asymptomatic polyps to symptomatic T3/T4 adenocarcinomas—supports a framework in which disease severity generally progresses with increasing depth of invasion and likelihood of lymph node involvement, while acknowledging the paradoxical presentations where large polyps may produce more pronounced symptoms than certain early adenocarcinomas [111].

### 6.3. Clinical Manifestations of Metastatic Colorectal Cancer (Stage IV Disease)

Accurate staging of CRC is essential for prognostication, therapeutic planning, and clinical management. The American Joint Committee on Cancer (AJCC) system remains the most widely used framework (https://ajccstaging.org last accessed on 8 September 2025), integrating tumor depth (T), regional lymph node involvement (N), and the presence of distant metastases (M) into stage groupings. Early stages (I–III) generally involve localized or loco-regional disease, with stage I encompassing T1–T2 lesions without nodal involvement, stage II describing T3–T4 lesions without nodal spread, and stage III representing any T lesion with regional lymph node metastases but without distant dissemination [112,113]. Stage IV CRC denotes the presence of distant metastases and is subdivided into IVA, IVB, and IVC, reflecting the extent and distribution of metastatic involvement. Stage IVA involves a single distant organ (e.g., liver or lung) or distant set of lymph nodes, IVB refers to multiple distant organs or nodal sites excluding peritoneal dissemination, and IVC indicates metastasis to the peritoneum, with or without additional organ involvement. The most frequent metastatic sites include the liver, lungs, regional and distant lymph nodes, and peritoneum, with hepatic involvement observed in approximately 50–60% of patients with stage IV disease, followed by lung metastases in 10–20%, peritoneal carcinomatosis in 10–15%, and nodal metastases in 5–10% [114,115,116]. Five-year survival rates progressively decline across stages: approximately 90–95% for stage I, 75–85% for stage II, 45–65% for stage III, and less than 15% for stage IV [112,113,114,115,116]. The T staging invites a deeper biological reflection that goes beyond the conventional N and M components, which, while prognostically relevant, offer limited insight beyond confirming more advanced disease. Indeed, positive nodal (N) or metastatic (M) status clearly delineates high-risk clinical scenarios. For instance, while a T2N0M0 tumor (stage I) generally carries an excellent prognosis compared to a T3N2M0 (stage III), a non-negligible proportion of T2N0M0 cases still relapse. This observation suggests that traditional morphology-based staging is incomplete and that future refinements should integrate molecular features capable of capturing the biological aggressiveness and recurrence potential of the disease.

Clinical manifestations of stage IV disease can be highly variable. A subset of patients remains asymptomatic despite significant metastatic burden, highlighting the insidious nature of CRC. Symptomatic presentations often correlate with the site of metastasis. Hepatic involvement may produce right upper quadrant discomfort, hepatomegaly, jaundice, fatigue, and laboratory abnormalities such as elevated liver enzymes. Pulmonary metastases can manifest as dyspnea, cough, or hemoptysis, whereas peritoneal dissemination may result in abdominal distension, ascites, early satiety, or sub-occlusive episodes. Metastatic lymph nodes can occasionally cause localized pain, palpable masses, or obstructive symptoms depending on their location. Non-specific systemic symptoms, including weight loss, anorexia, and fatigue, are also common and frequently precede organ-specific complaints. Compared to localized disease, the symptomatic spectrum of stage IV CRC is broader and often more complex, yet overlaps with earlier stages. For instance, patients may continue to experience rectal bleeding or altered bowel habits from the primary tumor while concurrently manifesting signs of distant organ involvement. Recognition of these patterns, in conjunction with imaging and biomarker evaluation, remains critical for timely initiation of systemic therapy and palliative interventions, aiming to optimize both survival and quality of life.

## 7. Poly-Metastatic and Oligo-Metastatic Colon Cancer: Need for Different Therapeutic Approaches

CRC exhibits a broad spectrum of metastatic behavior, ranging from limited metastatic spread (oligo-metastatic disease, OMD) to widespread dissemination (poly-metastatic disease). The distinction between these two states carries major therapeutic and prognostic implications, directly influencing the choice of treatment strategies and long-term outcomes. Therefore, before addressing systemic treatments for metastatic CRC, it is essential to acknowledge this biological and clinical dichotomy.

### Oligo-Metastatic Disease

**Figure 8:**
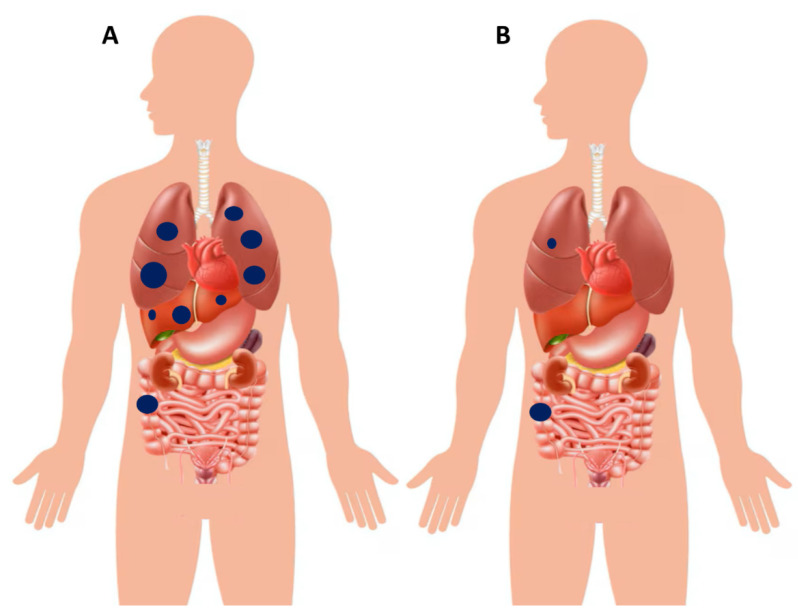
(**A**) Schematic illustration of a patient with right-sided colon cancer presenting with poly-metastatic disease, characterized by synchronous dissemination to the liver and lungs. (**B**) Example of another patient with right-sided colon cancer presenting with oligo-metastatic disease, limited to a single pulmonary lesion in the right lung. In this setting, upfront curative resection of the primary tumor combined with definitive local treatment of the metastatic lesion (e.g., surgery or stereotactic radiotherapy) is pursued. However, the role of post–definitive local therapy systemic chemotherapy is debatable, which may be reasonable in selected cases but still lacks confirmation from large randomized trials.

OMD is generally defined as the presence of a limited number of metastatic lesions, typically confined to one or two organs, most commonly the liver, lungs, or peritoneum. Examples of poly- and oligo-metastatic disease at diagnosis are schematically illustrated in Figure 8. Previous studies have provided a pragmatic and quantitative approach to defining OMD: specifically, oligo-metastases can refer to 1–3 metastatic tumors per organ with a maximum size of less than 7 cm [117]. More recently, a stricter definition has been proposed, encompassing 1–5 metastatic tumors with a maximum size of 5 cm [118]. An additional parameter that has been suggested is the “rate of metastatic growth,” which is usually slower in OMD, although it remains difficult to quantify and has limited applicability in routine clinical practice [119]. Beyond numerical or dimensional thresholds, other authors have emphasized a functional definition: OMD should be considered as the presence of metastatic cancer that is potentially amenable to curative or radical local interventions—such as surgery or radiotherapy—on all metastatic lesions, regardless of their number and/or volume [120,121]. Importantly, the American Society for Radiation Oncology (ASTRO) and the European Society for Radiotherapy and Oncology (ESTRO) consensus recently provided practical clinical definitions of OMD, underscoring the need for prospective validation and further scientific exploration [122]. Patients with OMD often display a more indolent biological trajectory compared with those with widespread disease, and this distinct clinical behavior opens the possibility of pursuing aggressive local strategies with curative intent. In this setting, surgical resection, SBRT (stereotactic body radiotherapy), and various ablative techniques represent cornerstones of management, frequently integrated with systemic therapies to optimize outcomes [123]. Among the different metastatic patterns, liver-limited disease is the most frequent presentation. Hepatic metastasectomy has long been established as a potentially curative option, and when combined with modern systemic therapy regimens, it can translate into substantial survival benefits, with five-year survival rates exceeding 50% in carefully selected patients [124,125]. A similar rationale applies to isolated pulmonary metastases. In patients with resectable lung lesions—particularly those harboring favorable molecular features, such as *RAS* and *BRAF* wild-type tumors—pulmonary metastasectomy has been shown to prolong survival and, in some cases, achieve long-term disease control [126,127]. Peritoneal involvement has traditionally been viewed as a hallmark of poly-metastatic disease, largely excluding patients from an OMD categorization. Nevertheless, this is not an absolute principle. In highly selected cases with limited peritoneal burden, cytoreductive surgery (CRS) combined with hyperthermic intraperitoneal chemotherapy (HIPEC) has emerged as a viable option. This multimodal approach has been associated with encouraging long-term survival outcomes, challenging the conventional perception that peritoneal metastases are invariably incompatible with an oligo-metastatic phenotype [128,129].

## 8. Systemic Treatments and the “Continuum of Care” Concept

### 8.1. General Considerations in Stages I–III and Liver-Limited Disease

Systemic therapy occupies a central role across the various stages of CRC, extending beyond advanced and metastatic disease into settings where the intent is curative. Its application varies according to tumor location (colon versus rectum), disease extent (localized, locally advanced, or metastatic), and biological characteristics (e.g., *RAS*/*BRAF* status, MSI-H/dMMR status). Stage I disease is primarily managed with surgery alone, as curative resection is usually sufficient without adjuvant systemic therapy. Standard surgical approaches include right or left hemicolectomy with complete mesocolic excision for colon cancer, and total mesorectal excision (TME) for rectal cancer, aiming to achieve adequate lymphovascular clearance and negative resection margins. Following surgical resection of both colon and rectal cancers—particularly stage III and high-risk stage II disease (features such as T4, insufficient lymph node sampling, poor differentiation, or lymphovascular invasion)—adjuvant systemic chemotherapy remains the standard of care. Fluoropyrimidines combined with oxaliplatin reduce the risk of recurrence and improve overall survival, whereas in lower-risk cases the intensity or duration of therapy may be reduced [130,131]. In this context, pre-treatment assessment for dihydropyrimidine dehydrogenase (DPYD/DPD) deficiency should be considered before administering fluoropyrimidine-based adjuvant regimens, since specific *DPYD* variants predict severe toxicity and allow genotype-guided dose reductions to improve safety [132]. However, the benefits of adjuvant chemotherapy must always be weighed against toxicity, especially neurotoxicity, and decisions should be individualized based on patient-specific risk factors [133]. Finally, the detection of minimal residual disease (MRD) by tumor-informed circulating tumor DNA (ctDNA) assays has emerged as a powerful prognostic biomarker and is increasingly studied to tailor adjuvant therapy: randomized and prospective studies have demonstrated that ctDNA-guided strategies can safely reduce overtreatment in stage II disease and identify patients at high risk of recurrence who may benefit from escalated adjuvant approaches [134,135].

For locally advanced rectal carcinoma (for example, clinical T3–T4 or node-positive disease), preoperative (neoadjuvant) therapy combining systemic agents and radiotherapy is employed to shrink tumors, increase the likelihood of R0 resection, and decrease local recurrence [136]. More recently, total neoadjuvant therapy (TNT)—delivering all systemic therapy upfront, often in combination with radiotherapy—has emerged as a promising strategy [137]. Its advantages lie in higher response rates, greater potential for downstaging, and improved opportunities for organ preservation, though challenges remain regarding timing, toxicity, and patient selection. In the setting of colorectal liver metastases, systemic therapy is applied in several contexts: to convert unresectable disease to resectable, to reduce tumor burden before surgery (neoadjuvant), and/or as adjuvant or perioperative therapy after resection. When metastases are resectable at baseline, systemic therapy may still reduce recurrence risk [138]. For borderline or initially unresectable disease, oxaliplatin- or irinotecan-based regimens, often combined with targeted agents, can achieve downstaging and allow curative surgery [139]. After resection, adjuvant or perioperative therapy reduces relapse risk, though consistent overall survival benefit is not always observed and depends on disease burden, patient selection, and tumor biology [123]. A paradigm shift has emerged in dMMR or MSI-H rectal cancers. In this subgroup, immune checkpoint inhibitors (ICIs) administered preoperatively have achieved extraordinarily high response rates, with some cases demonstrating complete regression. These results suggest the possibility of avoiding not only chemoradiation but, in selected cases, even surgery itself [140].

### 8.2. The “Continuum of Care” Paradigm

Systemic therapy plays an unquestionably central role in metastatic CRC, which must be understood as a continuum of care rather than a sequence of isolated treatment episodes. Patients follow a clinical trajectory defined by tumor biology, disease burden and distribution, treatment sensitivity, and fitness for successive interventions. Current ESMO guidance emphasizes three pillars for first-line decision-making: (i) upfront molecular stratification (extended *RAS*, *BRAF* V600E, mismatch repair/MSI status, *HER2* where relevant), (ii) patient-level fitness and symptoms, and (iii) organ-directed considerations. These elements determine whether the strategy is palliative systemic therapy, conversion therapy with curative intent, or immediate supportive care. As these initial decisions dictate the downstream continuum of care, they must be taken in the context of comprehensive biomarker testing and multidisciplinary review [6].

Two practical consequences follow. First, the radiological and biological response achieved with first-line therapy is one of the strongest prognostic indicators and is also predictive of benefit from subsequent lines. Measures such as early tumor shrinkage (ETS) and depth of response (DoR) correlate with progression-free, post-progression, and overall survival in modern trials. Patients achieving rapid and deep tumor regression are more likely to benefit from later treatments. This correlation is not merely statistical: the magnitude and timing of cytoreduction directly influence the feasibility of local consolidation (resection or ablation), the probability of durable control on subsequent regimens, and the maintenance of performance status after progression [141,142]. Second, the proportion of patients reaching third-line or later therapies has steadily increased. This reflects not only the availability of effective agents in later lines (oral multikinase inhibitors, nucleoside analogs, selective VEGFR inhibitors in some regions) but also advances in supportive care, toxicity management, and survivorship strategies that preserve patient fitness for additional therapies. As a result, the treatment paradigm for many patients is a staged strategy (induction→ consolidation/maintenance → salvage), with goals periodically re-evaluated according to disease course and patient condition [143].

Given the decisive impact of first-line treatment, several implications for practice arise: (a) rapid, comprehensive molecular profiling should be standard at metastatic diagnosis; (b) induction regimens should be selected for both potency and organ-preserving potential when conversion to surgery is a realistic goal; (c) sequencing should acknowledge that some targeted agents, such as anti-EGFR antibodies, achieve maximal efficacy in biomarker-selected subgroups and may be optimally employed early to maximize response depth; and (d) toxicity management and supportive care should be integrated from the outset to maximize eligibility for later lines.

### 8.3. First-Line Strategies in Poly-Metastatic Colorectal Cancer

First-line therapy in metastatic CRC has become increasingly individualized, guided by biomarkers, disease distribution, patient fitness, and therapeutic objectives. Treatment is generally based on combination chemotherapy doublets or triplets, to which targeted agents are added depending on molecular status. The strategy must balance the goal of maximal upfront control—particularly when conversion to resection is possible—against the need to preserve therapeutic options for later lines [6]. The standard chemotherapy backbones are FOLFOX (5-fluorouracil/leucovorin with oxaliplatin) and FOLFIRI (5-fluorouracil/leucovorin with irinotecan), considered broadly equivalent in efficacy but differing in toxicity profiles: oxaliplatin carries cumulative neurotoxicity, whereas irinotecan is associated with diarrhea and hematologic toxicity [144]. For selected fit patients with high tumor burden, the intensified triplet regimen FOLFOXIRI has shown superior efficacy compared with doublets, albeit with increased toxicity. The TRIBE and TRIBE2 trials validated FOLFOXIRI plus bevacizumab as a high-response induction option, particularly valuable in conversion settings [145,146].

Bevacizumab, targeting VEGF-A, remains the most widely used biologic in first-line irrespective of *RAS*/*BRAF* status. It improves progression-free survival and, in some studies, modestly overall survival, but carries risks including hypertension, proteinuria, impaired wound healing, and rare but serious vascular events [147,148]. Aflibercept, while more often used in later lines, illustrates the broader paradigm of sequential VEGF inhibition [149]. For *RAS* wild-type, left-sided tumors, anti-EGFR monoclonal antibodies (cetuximab or panitumumab) combined with chemotherapy have demonstrated superior outcomes compared with bevacizumab-based regimens. Landmark trials (FIRE-3, CALGB/SWOG 80405, PARADIGM) confirmed the predictive importance of both *RAS* status and tumor sidedness: left-sided, *RAS* wild-type tumors benefit most, while right-sided primaries respond less favorably [150,151,152]. The superior response of left-sided CRC to anti-EGFR agents stems from their higher prevalence of the canonical epithelial phenotype (CMS2), characterized by elevated expression of EGFR ligands (AREG, EREG), reduced immune and stromal interference, and consequently greater dependence on EGFR-driven signaling for proliferation and survival. In contrast, right-sided tumors frequently exhibit activation of alternative signaling pathways (WNT, PI3K, TGF-β, MAPK-independent growth) and a more inflamed microenvironment, both of which diminish their sensitivity to EGFR blockade [153,154]. Toxicities such as rash, hypomagnesemia, and infusion reactions are generally manageable, and cutaneous toxicity often correlates with efficacy.

A major paradigm shift has been the demonstration of immune checkpoint blockade efficacy in MSI-H/dMMR tumors. The KEYNOTE-177 trial established pembrolizumab as the new standard in this subgroup, with superior progression-free survival and favorable toxicity compared with chemotherapy. Nivolumab plus ipilimumab has also shown durable responses, primarily in previously treated patients [155,156]. Although MSI-H/dMMR tumors account for only 4–5% of metastatic CRC, the dramatic and durable benefit achieved underscores the need for universal testing at diagnosis. In some patients, immunotherapy achieves long-term control, reshaping the natural history of this subgroup [157].

### 8.4. Second-Line and Beyond in Poly-Metastatic Colorectal Cancer

Progression after first-line therapy represents a critical juncture in the continuum of care of metastatic CRC. Therapeutic choices are driven by prior drug exposure, molecular status, tumor sidedness, and performance status. A central principle is ensuring exposure to the three major cytotoxic agents (fluoropyrimidines, oxaliplatin, irinotecan) and at least one targeted class (anti-VEGF or anti-EGFR) during the treatment course [6]. For patients pretreated with oxaliplatin-based regimens, irinotecan-based therapy is standard in the second line, and vice versa. VEGF inhibition remains central: aflibercept with FOLFIRI (VELOUR trial) and ramucirumab with FOLFIRI both improved survival after oxaliplatin plus bevacizumab [148,158]. For *RAS* wild-type, left-sided tumors not exposed to anti-EGFR agents upfront, cetuximab or panitumumab can be introduced with irinotecan. Conversely, after prior anti-EGFR use, VEGF-targeted therapy is preferred. Acquired resistance mutations (e.g., *KRAS*, *NRAS*, *EGFR* extracellular domain) may regress after drug withdrawal, enabling anti-EGFR rechallenge strategies in later lines [159].

Beyond the second line, options have expanded considerably. Regorafenib, an oral multikinase inhibitor, improved survival in the CORRECT trial, though requires dose optimization due to toxicity [160]. Trifluridine/tipiracil (TAS-102), validated in the RECOURSE trial, confers survival benefit with manageable hematologic toxicity [161,162]. Both drugs provide similar benefit in refractory disease, and sequencing often depends on toxicity profiles [163].

The therapeutic landscape now also includes biomarker-driven strategies. *HER2* amplification (~3–5% of *RAS* wild-type metastatic CRC) predicts resistance to EGFR inhibitors but sensitivity to HER2-directed combinations [164]. *BRAF* p.V600E mutations (~5% of metastatic CRC) confer poor prognosis, but encorafenib plus cetuximab, with or without binimetinib, has become a standard later-line option [49,50]. *NTRK* fusions, though rare, are actionable with TRK inhibitors such as larotrectinib or entrectinib [165]. Finally, MSI-H/dMMR tumors not previously treated with ICIs remain sensitive even in later lines, with durable responses observed after multiple prior therapies [166].

In recent years, PIPAC (Pressurized IntraPeritoneal Aerosol Chemotherapy) has gained attention in the setting of peritoneal carcinomatosis from colorectal cancer, particularly beyond first-line systemic therapy [167]. PIPAC with oxaliplatin has been shown to induce objective tumor regression (histological response), stabilize quality of life, and confer survival benefit even in chemotherapy-refractory patients. In a phase I multicenter U.S. trial in heavily pretreated CRC/appendiceal cases, PIPAC was feasible and safe, with a median overall survival of ~12 months despite prior systemic treatment failure [168]. Moreover, PIPAC appears to modulate the tumor microenvironment toward reduced immunosuppression and may potentiate responses to immune checkpoint inhibitors [169]. Although randomized data are limited, PIPAC represents a promising locoregional adjunct in second-line and later settings for peritoneal disease when standard systemic options are exhausted.

### 8.5. Breaking the Therapeutic Line Paradigm: Rechallenge and Treatment Recycling Strategies in Metastatic Colorectal Cancer

A growing body of evidence has highlighted the feasibility and clinical relevance of rechallenge strategies in metastatic CRC. Rechallenge refers to the reintroduction of a therapy—cytotoxic, targeted, or immunotherapy—that was previously administered but discontinued due to disease progression or toxicity. The rationale stems from the dynamic nature of tumor evolution: under selective pressure, resistant clones may emerge during therapy, but over time, sensitive subclones can re-emerge, particularly after a drug holiday or exposure to alternative agents. This concept has been most extensively explored for anti-EGFR therapy, but also applies to certain cytotoxic agents and, increasingly, to molecularly targeted drugs.

Anti-EGFR monoclonal antibodies, such as cetuximab and panitumumab, are central in the treatment of *RAS* wild-type, left-sided CRC. Despite initial sensitivity, acquired resistance often arises, frequently mediated by emergent *RAS* or additional mutations [170]. However, molecular studies have shown that these resistant clones can decay over time once EGFR blockade is withdrawn, providing a window in which rechallenge may be effective [6,171]. Several prospective and retrospective studies have validated anti-EGFR rechallenge in carefully selected patients. The CRICKET trial demonstrated that patients with *RAS* wild-type metastatic CRC, previously treated with cetuximab and progressing on standard therapy, achieved an objective response rate of ~21% upon rechallenge. Importantly, the selection of candidates was guided by circulating tumor DNA (ctDNA) analysis, which allowed the identification of patients lacking detectable *RAS* or *EGFR* mutations at the time of rechallenge. This biomarker-driven approach underscores the value of real-time molecular monitoring to maximize efficacy while avoiding futile treatment [172]. Other trials, such as CHRONOS, have extended this concept, demonstrating that ctDNA-negative patients achieve meaningful responses to panitumumab rechallenge. These studies collectively support a paradigm in which anti-EGFR therapy is not irreversibly excluded after initial failure but can be strategically recycled in a molecularly informed manner [173]. Re-exposure to fluoropyrimidine-based or oxaliplatin-based regimens has also been explored. For instance, patients who previously received FOLFOX may benefit from FOLFOX reintroduction after a chemotherapy-free interval, particularly if prior treatment yielded a durable response and the patient retains adequate performance status. Clinical efficacy is generally lower than in the first exposure, but disease stabilization is achievable in a subset of patients, providing a bridge to further targeted therapies. Similarly, irinotecan-based regimens can be recycled with careful monitoring of cumulative toxicities, such as neuropathy for oxaliplatin or diarrhea for irinotecan [174,175]. Emerging evidence suggests that even molecularly targeted agents beyond anti-EGFR therapy might be considered for rechallenge. For example, in *HER2*-amplified metastatic CRC, dual HER2 blockade may be revisited following progression if intervening therapies allow re-sensitization, though this remains largely investigational. Similarly, BRAF V600E-directed therapy may be recycled in the context of combination strategies or after a drug holiday, contingent on patient tolerance and molecular evolution [176,177].

Some clinical considerations can be pragmatically shared. In fact, rechallenge strategies are generally reserved for fit patients with preserved organ function and good performance status, and they are most effective when there has been a meaningful interval since prior exposure. Toxicities from previous lines must be carefully weighed, particularly cumulative neuropathy from oxaliplatin or hematologic toxicity from repeated fluoropyrimidine exposure. Importantly, rechallenge does not replace the need for conventional sequencing of systemic therapies but represents a complementary strategy to extend the continuum of care and optimize long-term outcomes.

Beyond clinical efficacy and toxicity, modern oncology increasingly recognizes the importance of integrating patient-reported outcomes (PROs) and quality of life (QoL) measures into therapeutic decision-making. These parameters provide a direct insight into patients’ subjective experiences, functional well-being, and the cumulative burden of treatment over successive lines of therapy. In metastatic CRC, where disease control and survival are often balanced against chronic toxicity and treatment fatigue, incorporating PROs allows for a more patient-centered interpretation of the “continuum of care.” In this perspective, attention must also be directed toward understanding how treatment-related variables concretely affect patients’ perceived well-being and daily functioning. In fact, several studies have shown that treatment intensity, cumulative neurotoxicity, fatigue, and gastrointestinal side effects substantially influence long-term QoL, sometimes outweighing survival gains [178,179]. Consequently, therapeutic sequencing should not only be guided by biological efficacy but also by the preservation of autonomy, social functioning, and psychological resilience. The integration of structured PRO and QoL assessments into clinical trials and real-world practice thus represents a crucial evolution toward a truly holistic management of metastatic CRC, aligning therapeutic ambition with the lived experience of patients [180].

## 9. Methodological Challenges in Colorectal Cancer Research

### 9.1. Studying Intermittent Therapy: An Urgent Need but Also a Formidable Methodological Challenge

#### 9.1.1. Studying Intermittent Therapy: Rationale and Definitions

In metastatic CRC eligible for anti-EGFR therapy, the combination of chemotherapy with anti-EGFR monoclonal antibodies, such as cetuximab or panitumumab, has demonstrated substantial efficacy and represents a cornerstone of current therapy [6]. However, prolonged administration is frequently associated with toxicities (often cumulative)—including dermatologic, gastrointestinal, and neurologic adverse events—that can negatively affect body image, self-perception, physical and emotional functioning, and overall quality of life [181,182,183]. To mitigate these drawbacks while maintaining disease control, intermittent treatment strategies have been explored [184,185,186]. These approaches alternate periods of active therapy with treatment breaks, aiming to preserve efficacy while reducing the toxicity burden. Importantly, intermittent therapy refers specifically to regimens in which all systemic agents are completely withdrawn during planned breaks in patients achieving objective response or stable disease, with no maintenance or de-escalated therapy administered in interim. Although the aim of preserving efficacy while reducing toxicity and respecting quality of life is similar, protocols that replace full interruption with maintenance monotherapy (e.g., single-agent fluoropyrimidine and/or biologic) constitute treatment de-escalation rather than true intermittence and are therefore beyond the scope of this methodological discussion.

#### 9.1.2. Methodological Challenges and Biological Considerations

Although conceptually appealing, intermittent strategies are undermined by methodological limitations in currently published clinical trials, compromising both their clinical applicability and biological interpretability. Table 3 summarizes the key methodological characteristics of major trials investigating true intermittent anti-EGFR-based strategies in mCRC.

A critical concern is that, whether designed as superiority or non-inferiority, intermittent-therapy trials inevitably derive their hypotheses and effect-size assumptions from continuous-treatment studies, despite substantial differences in treatment objectives, selective pressures, and resistance dynamics. The COIN-B and IMPROVE trials, for example, established their hypothesis benchmarks partly based on earlier studies lacking anti-EGFR therapy [MRC FOCUS [187] and the control arm of CRYSTAL [188], respectively], whereas PRODIGE-28 defined statistical efficacy thresholds without explicitly referencing prior trials. In some cases, these benchmarks appear overly conservative—for instance, a 6-month progression-free survival (PFS) rate of 40% or a median PFS on treatment (PFSot) of 7 months—compared with contemporary data showing >8 months median PFS with FOLFIRI (folinic acid + fluorouracil + irinotecan) plus anti-EGFR therapy in similar settings [150]. Notably, older data from COIN-B, reporting a 10-month failure-free survival of 50%, may more accurately reflect the efficacy of chemotherapy plus anti-EGFR combinations [149,165,189].

These limitations are compounded by the fact that intermittent-therapy protocols are governed by fixed or calendar-based schedules rather than real-time assessments of tumor biology. Although fixed schedules can simplify operational logistics, they forego opportunities to incorporate dynamic biomarkers that could guide treatment interruption or re-starting according to emerging resistance mechanisms. In the above cited trials, treatment discontinuation was performed without concurrent molecular monitoring of tumor evolution. The absence of real-time genetic data limits treatment adaptation to evolving tumor biology and may delay optimal therapeutic decisions. Disease progression during treatment-free intervals should not be assumed to occur without the emergence of *RAS*-mutated resistant clones. Indeed, in intermittent therapy, clonal evolution may continue during treatment breaks [171,190]. If *RAS* mutations do not arise, anti-EGFR rechallenge can remain effective; if they do, delayed detection may postpone the beginning of an alternative regimen, prolonging ineffective treatment. Serial liquid biopsy monitoring—particularly for *RAS*/*BRAF* mutations and other resistance-associated alterations—could enable earlier therapeutic switching and preserve subsequent treatment efficacy [191]. Incorporating such molecular assessments into trial design would enhance reliability, robustness, and alignment with principles of tumor evolutionary biology. Beyond improving individual trial methodology, large, shared real-world datasets could be leveraged to study the *RAS* mutational dynamics during or after treatment interruption—a “time-to-molecular progression” parameter not yet investigated in intermittent studies. Embedding biomarker-driven decision rules would benefit from access to harmonized, therapy-specific large datasets. In this context, the European Alliance for Personalized Medicine (EAPM) has underscored the urgent need for multinational sharing of all clinical and molecular oncology data, paralleling ongoing initiatives in the United States [192]. Such collaboration would allow pooling of intermittent-therapy-specific real-world evidence, strengthening both statistical bases and biological interpretability of future trials.

This limitation also underscores a broader challenge in oncology: the scarcity of robust clinical-predictive molecular markers for both targeted therapies and conventional cytotoxic agents. During anti-EGFR therapy, one of the most frequent mechanisms of tumor genetic evolution leading to resistance is the emergence of *RAS* mutations. However, since anti-EGFR agents are almost always combined with other systemic therapies, broader selective pressures act on pathways beyond *RAS*, fostering the parallel development of multiple resistance mechanisms. In this setting, additional genomic alterations commonly co-occur, sustaining a diverse spectrum of resistance phenotypes [170]. These observations indicate that *RAS* mutation detection during therapy should be interpreted not solely as a marker of anti-EGFR resistance but as part of a broader co-evolutionary process that may influence the efficacy also of other agents included in the combination regimens. Notably, the primary chemotherapeutic backbone in metastatic CRC comprises fluorouracil, irinotecan, and oxaliplatin, administered in both continuous and intermittent schedules. Among these, oxaliplatin exerts broad cytotoxic effects by inducing bulky DNA adducts and double-strand breaks [193]. In contrast, fluorouracil and irinotecan act through defined molecular targets. Fluorouracil irreversibly inhibits thymidylate synthase (TYMS), leading to thymidine depletion and impaired DNA synthesis; accordingly, its activity is modulated by TYMS expression [194]. Irinotecan, a prodrug converted into the active metabolite SN-38, mediates cytotoxicity through potent inhibition of topoisomerase I (Topo-I) [195]. Resistance to these agents may arise through TYMS gene amplification [196] or Topo-I mutations [197], which can co-occur with *RAS*-driven resistance to anti-EGFR therapy. These molecular alterations could provide a strong rationale for specifically investigating strategies of longitudinal monitoring and potential therapeutic adaptation, with the ultimate aim of reducing empiricism when reintroducing cytotoxic chemotherapy after progression in intermittent schedules.

Biological heterogeneity additionally complicates this interpretation. Left- versus right-sided *RAS*/*BRAF* wild-type CRC exhibit distinct prognostic and predictive profiles under anti-EGFR therapy. Right-sided tumors often display aggressive molecular features and a rapid emergence of resistance mechanisms, not limited to *RAS* mutations but also involving alterations in *RAF*, *MEK*, and other downstream effectors, raising concern that treatment holidays could disproportionately compromise disease control [198,199]. In the trials considered, right-sided tumors comprised 31% (COIN-B), 27% (PRODIGE-28), and 16% (IMPROVE) of participants, reflecting heterogeneity of clinical decisions for these patients and population variability. Eligibility criteria, stratification factors, and endpoint definitions should account for such differences to yield reliable conclusions. Thus, intermittent anti-EGFR trials are limited by fixed treatment schedules, and lack of real-time molecular monitoring, with heterogeneous populations and variable endpoints restricting cross-trial comparability.

#### 9.1.3. Endpoints and Future Directions in Precision Intermittent Therapy

Endpoint selection represents an additional methodological challenge. Conventional PFS, while well established, does not fully capture clinical outcomes in patients undergoing planned treatment breaks. Modified metrics, such as PFSot, measure time on treatment until progression, including retreatment after interval progression, but can overestimate benefit, particularly when progression is documented radiologically only after therapy resumption. Furthermore, the PFSot endpoint exemplifies how endpoint definitions may interact with biological variables. By design, PFSot extends beyond conventional PFS by including continued therapy after progression during treatment-free intervals. This can be misleading in cases of early resistance, where cumulative time—including treatment interruption, radiologic confirmation, retreatment, and second progression—artificially inflates PFSot without reflecting a clinical benefit. Such artifacts may partially explain the IMPROVE trial findings in left-sided tumors, where intermittent therapy arm B showed a median PFSot of 23.9 months versus 11.7 months for continuous therapy, without a survival benefit (35.9 vs. 36.2 months). Conversely, in right-sided tumors, intermittent therapy was associated with shorter survival (24.9 vs. 37.7 months) and lower PFSot (7.8 vs. 10.1 months), consistent with accelerated RAS-mutation emergence in this subgroup.

The current evidence on intermittent anti-EGFR–based strategies in metastatic CRC is constrained by methodological misalignment with the biology they are meant to exploit more than by a lack of efficacy. Fixed, schedule-driven designs, outdated benchmarks, and endpoints insensitive to clonal dynamics risk obscuring true benefit–risk profiles and limiting generalizability. The incorporation of serial molecular monitoring and biology-adapted decision rules into trial design should no longer be regarded as ancillary, but rather as a methodological requirement essential for the correct interpretation of results. Only by aligning therapeutic timing with tumor evolution can intermittent therapy progress from an appealing concept to a precision tool capable of improving patient outcomes.

### 9.2. The Role of Circulating Tumor DNA and Variant Allele Frequency in Precision Oncology of Colorectal Cancer

#### 9.2.1. Clinical Utility of ctDNA Across CRC Stages

Circulating tumor DNA (ctDNA) has emerged as a significant prognostic biomarker in CRC, offering insights into minimal residual disease (MRD), treatment response, and disease progression across clinical stages; unlike circulating tumor cells (CTCs), ctDNA analysis is technically simpler, more reproducible, and readily amenable to serial monitoring [200]. Recent studies have underscored its utility in both the adjuvant and metastatic settings. In the adjuvant setting, ctDNA analysis has demonstrated its prognostic value. A pivotal study by Tie et al. revealed that the presence of ctDNA after curative surgery in stage II CRC patients was associated with a markedly increased risk of recurrence, whereas its absence indicated a low risk, suggesting that ctDNA could guide decisions regarding adjuvant chemotherapy [201]. In metastatic CRC, ctDNA serves as a valuable tool for monitoring disease progression and treatment efficacy. Taïeb et al. demonstrated that early ctDNA variation could predict progression-free and overall survival in patients with dMMR/MSI-H metastatic CRC treated with ICIs [202]. Additionally, a recent study suggested that ctDNA could help identify patients who might benefit from a therapy switch before confirmation by radiologic imaging [203]. The ROME trial provides important evidence for the clinical help of integrating tissue and liquid biopsies in precision oncology, particularly emphasizing the prognostic value of concordant genomic alterations detected across both modalities [204].

However, one critical issue that remains insufficiently explored in these studies is the role of variant allele frequency (VAF) in shaping the prognostic and predictive interpretation of genomic profiling.

#### 9.2.2. Variant Allele Frequency as a Surrogate of Clonal Dominance

VAF is not merely a technical readout of sequencing; it represents a surrogate measure of the biological dominance of a given alteration within the tumor ecosystem [205]. A high VAF indicates that a mutation extends beyond a minor subclone and is diffusely represented across tumor cells. Conversely, a low VAF may reflect subclonal events, passenger mutations, or technical detection limits [206]. Therefore, when concordant alterations are observed in both tissue and plasma, the associated survival benefit may reflect not only “double detection,” but also the underlying clonal prevalence and dominance of these alterations manifesting across compartments.

On this light, VAF provides a biologically based explanation for why concordant alterations carry stronger prognostic weight: they represent clonal driver mutations that shape tumor biology and are readily detectable across platforms [206,207]. On the other hand, discordant findings may originate from low-VAF subclones confined to specific tumor niches or variably shed into circulation. Such alterations, while potentially actionable, may exert less influence on global tumor behavior and thus confer weaker prognostic or therapeutic relevance. Therefore, incorporating VAF into concordance analyses could refine the interpretation of clinical outcomes. Rather than a binary definition of concordance (present/absent), a quantitative, VAF-adjusted framework could more accurately capture the biology underpinning clinical benefit. Stratifying concordant cases by VAF levels, or adjusting hazard models for clonal representation, may help to clarify whether survival differences reflect assay concordance or the clonal architecture of the tumor.

Consideration of VAF also aligns with the broader shift in genomic oncology toward quantitative rather than binary biomarkers. Much like tumor mutational burden (TMB) or circulating tumor fraction, VAF conveys both technical and biological meaning. This is particularly relevant in liquid biopsy, where detection thresholds and shedding heterogeneity complicate interpretation. In this setting, VAF can help into discrimination of biologically dominant alterations from background “noise”.

#### 9.2.3. Technical and Methodological Considerations in ctDNA Analysis

Another methodological aspect warranting attention is the temporal dimension of sampling. While the ROME trial accepts archival tissue collected up to six months before enrolment, liquid biopsy provides a real-time snapshot. Therefore, discordance between the two may reflect true tumor evolution rather than analytical limitations. Here again, VAF can bridge the gap: a rising VAF in plasma may indicate emerging clonal sweeps or therapy-driven selection, serving as a dynamic readout of tumor adaptation.

The extension of liquid biopsy to alternative biofluids (e.g., cerebrospinal fluid, pleural effusion, ascitic fluid) additionally defines the value of VAF as a unifying quantitative marker. In such compartmentalized settings, VAF could harmonize interpretation across fluids and platforms. Equally important, preanalytical variables such as specimen collection, storage, and shipment must be recognized as potential sources of variability. As an example, in the ROME trial, plasma samples were processed centrally at the FoundationOne facility in Europe. Although centralization minimizes inter-assay variability, long-distance shipment introduces risks for ctDNA quality. Blood was likely collected in Roche Cell-Free DNA collection Tubes^®^, which preserve specimens for up to seven days, though stability is influenced by temperature and handling. Indeed, Roche tubes demonstrate superior short-term preservation compared with Streck Cell-Free DNA BCT^®^ at room temperature, though their performance declines beyond seven days [208]. These technical considerations must be considered when interpreting tissue–liquid concordance.

Specifically, pre-analytical factors such as the type of collection tube, time-to-processing, and plasma volume exert measurable effects on ctDNA yield and variant allele frequency. Delayed plasma separation (>4–6 h in standard EDTA tubes) promotes leukocyte lysis and dilution of ctDNA with genomic DNA, thereby reducing apparent VAF and assay sensitivity [209]. Specialized preservative tubes (e.g., Streck, Roche) mitigate this effect by stabilizing nucleated cells and circulating DNA fragments for several days, yet temperature excursions or extended storage still compromise integrity. Likewise, inadequate plasma volume—often <4 mL per 10 mL of whole blood—can lead to insufficient total cfDNA input, disproportionately impacting low-VAF variant detection [210]. The cumulative consequence is a potential underestimation of tumor fraction and false-negative results, particularly in minimal residual disease contexts. To minimize these biases, clinical studies and laboratories should enforce standardized pre-analytical protocols, including the use of validated preservation tubes, immediate or timely centrifugation within defined time limits, controlled-temperature transport, and collection of adequate plasma volume (ideally ≥8–10 mL). Such measures enhance reproducibility and ensure that observed differences in ctDNA yield or VAF reflect true biological variability rather than pre-analytical artifacts.

In conclusion, future analyses should systematically incorporate VAF, pre-analytical factors, and disease- and therapy-specific stratification into their interpretive framework. Recognizing VAF as a surrogate marker of clonal dominance may enable more accurate patient selection for targeted therapies and enhance the predictive value of molecular tumor boards.

### 9.3. Refining Patient Selection: Challenges and Opportunities

The mission of refining patient selection is more critical than ever, particularly in metastatic settings. We now have more sophisticated molecular tools and deeper biological insights to identify patients most likely to benefit from specific therapies. It is well established that molecular assessment (e.g., extended *RAS* and *BRAF* mutational profiling) remains a cornerstone of therapeutic decision-making [6]. Moreover, the recognition that MSI-H status predicts responsiveness to ICIs has arguably transformed the therapeutic paradigm for this subset. Nevertheless, significant challenges persist in further optimizing patient selection [153,154,155,156,157].

One emergent concept is that of negative hyper-selection: beyond the classical *RAS*/*BRAF* wild-type status, additional genomic alterations (e.g., *HER2* or *MET* amplifications, *MAP2K1* mutations) may actively drive resistance to EGFR monoclonal antibodies, and their exclusion can sharpen the predictive value of selection. In fact, in recent studies almost 50% of *RAS*/*BRAF* wild-type tumors harbor additional resistance alterations detectable by ctDNA, arguing for a more granular “exclusion profiling” in first-line anti-EGFR therapy. This approach helps avoid patient exposure to ineffective agents and guides more personalized therapy allocation [170].

However, beyond genomics, the TME remains a largely untamed frontier of selection. The interplay of immune cells, stromal fibroblasts, extracellular matrix, and metabolic constraints can alter drug penetration, immune surveillance, and resistance. The elusive “immune competence” of the TME—both baseline and under treatment pressure—may meaningfully modulate therapeutic efficacy, yet we lack robust, clinically operative biomarkers that capture this complexity [211]. Equally challenging is the dynamic heterogeneity of metastatic disease: clones evolve under therapeutic pressure, subclones wax and wane, and the static snapshot from a primary biopsy may misrepresent the evolving mutational landscape in metastatic lesions or via ctDNA. The evolving use of liquid biopsy seeks to monitor real-time change, but standardization and longitudinal integration remain works in progress [212].

One illustrative example of granular patient stratification lies in antibody-dependent cellular cytotoxicity (ADCC). Some monoclonal antibodies—especially those engineered for enhanced Fc effector function—exert part of their antitumor activity via NK cell–mediated ADCC [213]. It has been proposed that patients with the FcγRIIIA *v/v* polymorphism, which binds IgG1 antibodies with higher affinity, could derive greater benefit from such agents. This concept exemplifies how immunogenetic stratification might refine monoclonal antibody therapy [214,215].

Similarly, the “granularity” of *KRAS* mutations is increasingly recognized: not all *KRAS* alterations have equal prognostic or predictive weight [26,27]. In fact, the emergence of *KRAS* p.G12C inhibitors in other tumor types raises the possibility—even in CRC—of targeted therapy for a defined subpopulation, further underscoring the need for mutation-level granularity [216].

In sum, we stand at a juncture where the promise of precision medicine in metastatic CRC must evolve into hyper-selection: not merely selecting what is positive, but rigorously excluding what portends resistance. To fulfill this promise, our clinical algorithms must integrate (i) expanded negative molecular profiling, (ii) assessments of TME and immune competence, (iii) longitudinal monitoring of clonal dynamics, and (iv) functional immunogenetic stratifiers such as Fc receptor polymorphisms. Only by weaving these layers of information can we approach the goal—and the duty—of optimally matching each patient to the therapy most likely to succeed.

## 10. Other Unresolved Challenges and Future Directions

Identifying and summarizing the key challenges in CRC research and management is crucial for guiding future investigations and informing clinical practice. Despite significant advances in early detection, therapeutic strategies, and precision medicine, several critical barriers continue to limit the translation of scientific progress into improved patient outcomes. Recognizing these unresolved issues allows researchers and clinicians to prioritize efforts, design rational interventions, and develop evidence-based strategies that address both biological complexities and healthcare disparities. By systematically outlining these challenges, the field can accelerate innovation, optimize patient stratification, and ultimately improve survival and quality of life for patients with CRC. Four major challenges can be highlighted.

### 10.1. Early Detection and Prevention

Early detection remains a top priority. Although colonoscopy and fecal-based screening tests have reduced CRC mortality, adherence remains suboptimal [217]. Lowering the age for the first colonoscopy could further improve early detection rates, particularly in populations with increasing incidence among younger adults. In parallel, novel and minimally invasive approaches, such as liquid biopsy (ctDNA-based detection) and AI-driven risk stratification models, hold promise for enhancing early diagnosis and identifying high-risk populations.

### 10.2. Overcoming Therapeutic Resistance

Primary and acquired resistance to targeted therapies and immunotherapy continues to represent a major obstacle. Mechanistic studies investigating tumor plasticity, epigenetic reprogramming, and clonal evolution are critical for the development of next-generation inhibitors and rational drug combinations capable of overcoming resistance.

### 10.3. Expanding the Role of Immunotherapy

While ICIs have transformed the management of dMMR/MSI-H tumors, the majority of MSS/pMMR CRC cases remain refractory. Strategies aimed at converting immunologically “cold” tumors into “hot” tumors—such as combination approaches with STING (Stimulator of Interferon Genes) agonists, Interleukin-2-based immunomodulation, or microbiome engineering [218,219,220]—represent a promising frontier in immunotherapy. However, recent late-phase clinical development has increasingly emphasized rational immunotherapy-based combinations designed to overcome the intrinsic resistance of microsatellite-stable (MSS) or proficient mismatch repair (pMMR) colorectal cancers to immune checkpoint inhibition [221,222,223,224,225]. The guiding rationale of these approaches is twofold: to enhance intratumoral immune priming and T-cell trafficking, and to dismantle the stromal and angiogenic programs that sustain immune exclusion and blunt checkpoint efficacy.

Among the most clinically advanced strategies, combinations of ICIs with multi-kinase tyrosine kinase inhibitors (TKIs) such as regorafenib or cabozantinib have shown encouraging early signals of activity in otherwise refractory MSS tumors [221,222,223]. These agents act by reprogramming the tumor microenvironment—reducing VEGF-driven immunosuppression, altering tumor-associated macrophage (TAM) polarization, and normalizing vasculature—thereby enhancing immune cell infiltration and checkpoint sensitivity. Parallel efforts have evaluated the integration of ICIs into cytotoxic and anti-angiogenic backbones. In the AtezoTRIBE randomized phase II trial, the addition of atezolizumab to the FOLFOXIRI plus bevacizumab regimen improved outcomes in immune-enriched molecular subsets, suggesting that transient cytotoxic debulking coupled with VEGF blockade may transiently remodel the tumor microenvironment, promoting immune recognition and infiltration [224]. Similarly, the combination of lenvatinib, a potent inhibitor of VEGFR, FGFR, and MAPK signaling, with pembrolizumab is being investigated in the phase III LEAP-017 trial for previously treated pMMR/MSS mCRC. This regimen aims to synergize angiogenic and stromal modulation with PD-1 blockade to restore immune responsiveness in otherwise “cold” tumors [225].

Collectively, these trials signal a paradigm shift from empiric combinatorial immunotherapy to mechanistically informed regimens that specifically target tumor microenvironment components—angiogenesis, macrophage polarization, extracellular matrix remodeling, and MET signaling—that underlie immune exclusion. Notably, accumulating evidence suggests that the therapeutic benefit of these combinations is concentrated in molecularly or microenvironmentally defined subgroups, such as patients lacking extensive liver metastases or exhibiting favorable Immunoscore-IC or tumor-intrinsic immune signatures [10,226]. These findings underscore the need for integrated translational endpoints in ongoing phase III programs to identify patients most likely to achieve genuine conversion from “cold” to “hot” tumor phenotypes under combinatorial immunotherapy.

### 10.4. Bridging Disparities in Colorectal Cancer Outcomes

Socioeconomic factors continue to influence CRC incidence and survival, with significant racial and geographic disparities in access to screening and molecular testing [227]. Expanding precision medicine initiatives beyond major academic centers is essential to ensure equitable healthcare delivery and improve outcomes across diverse populations.

## 11. Conclusions

CRC is a highly heterogeneous disease, shaped by a complex interplay of genetic, epigenetic, and environmental factors. Over the past decades, advances in molecular profiling have transformed our understanding of its biology, enabling more effective screening strategies, refined prognostic tools, and personalized therapeutic approaches. Despite these considerable achievements, significant challenges persist, and the path toward fully overcoming the disease remains long and complex. Nonetheless, the integration of multidisciplinary care, combined with progressively deeper mechanistic and methodological insights, is equipping clinicians and researchers with increasingly sophisticated tools to enhance early detection, optimize therapeutic strategies, and ultimately improve survival, disease control, and quality of life for patients across all stages of the disease.

## Figures and Tables

**Table 2:** Age-standardized incidence rates of colorectal cancer by region, with colon-to-rectal ratios and epidemiological trends (2022).

Region/Continent	CRC Incidence (Colon + Rectal) ASR/100,000 (Both Sexes)	Colon/Rectal Ratio	Key Notes and Trends
Worldwide	~18.4	2.0	Global colon cancer incidence is approximately double rectal; overall CRC incidence rising modestly worldwide. EOCRC (<50 years) increasing in high-income countries and emerging in low/middle-income countries.
Australia/New Zealand	~35	1.6	Among the highest CRC ASIR globally. Screening and early detection are established; colon vs. rectal cancer both high; some decline in older age incidence; rise in younger.
Europe (Western/Northern)	~30–45 in many high-rate countries (e.g., Denmark ~48.1, Norway ~45.3, Netherlands ~42.8)	2.0	High rates for colon cancer; rectal cancer also high. Some Western European countries show stable or declining colon cancer incidence in older age groups; rectal incidence among younger increasing.
North America (U.S., Canada)	~37.1	2.3	U.S., Canada show declines in older adults but strong increases among younger, especially for rectal cancer. Distinctions in colon vs. rectal trends visible.
East Asia	China ~20.1 Japan ~36.6	2.5	East Asia has among highest absolute numbers due to large population; both colon and rectal cancer incidence rising. EOCRC burden increasing.
Latin America/Caribbean	~18–25	2.5	Lower ASIR, but increasing trends; colon cancer rising as lifestyle changes; rectal cancer also contributes significantly.
South/South-Central Asia	~4.9	2.5	Underreporting may be an issue; less screening; incidence rising slowly but mortality high because of late diagnosis.
Sub-Saharan Africa	<10	3.0	Countries with increasing obesity and diet shifts have rising CRC (colon + rectum); projections expect increases. Some nations show divergence where rectal cancer rises faster in younger cohorts.

**Table 3:** Methodological characteristics of phase II trials evaluating intermittent anti-EGFR-based strategies in *RAS* wild-type metastatic CRC.

Study	Sample Size	Induction Regimen	Study Arms	Primary Endpoint	Statistical Hypothesis	Biomarker-Guided Stop?	Study Conclusions
COIN-B	169	12 weeks FOLFOX + cetuximab	Randomization after induction: intermittent FOLFOX/cetuximab vs. intermittent FOLFOX + cetuximab maintenance (reintroduction of FOLFOX at progression)	FFS	A’Hern’s design to detect a 10-month FFS of 50% versus 35%, based on prior MRC FOCUS and phase II trial; 80% power and 5% one-sided α	No	The trial did not meet its primary endpoint; intermittent chemotherapy with cetuximab maintenance was feasible but failed to demonstrate superiority in FFS
PRODIGE-28	139	8 cycles FOLFIRI + cetuximab	Randomization after induction: intermittent FOLFIRI/cetuximab vs. intermittent FOLFIRI + cetuximab maintenance (reintroduction of FOLFIRI at progression)	PFR	Fleming one-step design with a 6-month PFS rate of 40% as null hypothesis; 80% power and 5% one-sided α	No	The study showed activity of induction plus cetuximab but did not achieve the predefined efficacy threshold; intermittent treatment did not improve PFR compared to continuous therapy
IMPROVE	137	8 cycles FOLFIRI + panitumumab	Randomization after induction: continuous FOLFIRI + panitumumab until progression vs. intermittent strategy with planned treatment-free intervals	PFSot	Hypothesis based on lower 95% CI of CRYSTAL study, median PFSot time ≤7 months as null hypothesis; 80% power and 10% one-sided α	No	The trial met its statistical design but provided no evidence that intermittent strategies with panitumumab were superior; clinical benefit remained comparable to continuous therapy

CI: Confidence interval; COIN-B: Continuous or intermittent chemotherapy in combination with cetuximab—B arm; CRYSTAL: Cetuximab combined with irinotecan in first-line therapy for metastatic colorectal cancer; EGFR: epidermal growth factor receptor; FFS: Failure-free survival; FOLFIRI: Folinic acid + fluorouracil + irinotecan; FOLFOX: Folinic acid + fluorouracil + oxaliplatin; IMPROVE: Intermittent or continuous panitumumab plus fluorouracil, leucovorin, and irinotecan for first-line treatment of *RAS* and *BRAF* wild-type metastatic colorectal cancer; MRC FOCUS: Medical research council fluorouracil, oxaliplatin, and CPT-11 use and sequencing trial; PFR: Progression-free rate: PFR; PFS: Progression-free survival; PFSot: Progression-free survival on treatment; PRODIGE 28: Partenariat de recherche en oncologie digestive 28.

## Data Availability

No new data were created or analyzed in this study.
